# Circulating Cytokines and Coronavirus Disease: A Bi-Directional Mendelian Randomization Study

**DOI:** 10.3389/fgene.2021.680646

**Published:** 2021-06-07

**Authors:** Mengyu Li, Chris Ho Ching Yeung, C. Mary Schooling

**Affiliations:** ^1^School of Public Health, Li Ka Shing Faculty of Medicine, The University of Hong Kong, Hong Kong, China; ^2^Graduate School of Public Health and Health Policy, City University of New York, New York, NY, United States

**Keywords:** cytokine, coronavirus disease, Mendelian randomization, genetics, immune system

## Abstract

**Background:**

Immune system functioning is relevant to vulnerability to coronavirus disease (COVID-19). Cytokines are important to immunity. To further elucidate the role of the immune system in COVID-19, we used Mendelian randomization (MR) to assess comprehensively and bi-directionally the role of cytokines in COVID-19.

**Methods:**

We assessed primarily whether genetically different levels of 41 cytokines affected risk of any COVID-19 (laboratory confirmed, physician confirmed or self-reported, 36,590 cases, 1,668,938 controls), and conversely if genetic risk of liability to any COVID-19 affected these cytokines (*n* ≤ 8293) using the most recent genome-wide association studies. We obtained inverse variance weighting (IVW) estimates, conducted sensitivity analyses and used a Benjamini-Hochberg correction to account for multiple comparisons. We also assessed whether any findings were evident for hospitalized COVID-19 (hospitalized laboratory confirmed, 12,888 cases, 1,295,966 controls).

**Results:**

Macrophage inflammatory protein-1β (MIP1b; more commonly known as Chemokine (C-C motif) ligands 4 (CCL4) was inversely associated with COVID-19 [odds ratio (OR) 0.97 per SD, 95% confidence interval (CI) 0.96–0.99] but not after adjustment for multiple comparisons. This finding replicated for hospitalized COVID-19 (OR 0.93, 95% CI 0.89–0.98). Liability to any COVID-19 was nominally associated with several cytokines, such as granulocyte colony-stimulating factor (GCSF) and hepatocyte growth factor (HGF) but not after correction.

**Conclusion:**

A crucial element of immune response to infection (CCL4) was related to COVID-19, whether it is a target of intervention to prevent COVID-19 warrants further investigation.

## Background

Coronavirus disease (COVID-19) caused by the severe acute respiratory syndrome coronavirus 2 (SARS-CoV-2) is a serious global pandemic^1^. Vulnerability to and response to infection with SARS-CoV-2 varies widely^2^ underscoring the role of the immune response, which is regulated by cytokines (Kany et al., 2019). A “cytokine storm” may occur in severe COVID-19 and results in worse prognosis (Chen et al., 2020; Huang et al., 2020; Sun et al., 2020). Several trials of anti-inflammatory medications, such as tocilizumab and sarilumab targeting the interleukin (IL)-6 receptor, and anakinra, i.e., targeting IL-1 receptor antagonism, are underway^3^ or completed^4,5,6,7,8^ (Stone et al., 2020; Hermine et al., 2021; Salvarani et al., 2021). A recent large trial suggested mortality benefits of tocilizumab in some groups of hospitalized patients^7^, consistent with a previous Mendelian randomization (MR) study suggesting IL-6 blockade might be helpful in COVID-19 hospitalization (Bovijn et al., 2020). In contrast, the wider role of cytokines in COVID-19 has been less investigated, using MR to compare COVID-19 outcomes in people with genetically predicted different levels of cytokines. MR gives less-confounded estimates by making use of the randomization of genetic variants at conception, which is similar to the randomization in randomized controlled trials (RCTs), and has foreshadowed findings from trials^9^ (Lawlor et al., 2008). MR has previously been used successfully to investigate potential targets for COVID-19 prevention (Leong et al., 2020; Ponsford et al., 2020). Here, we used MR to investigate whether any of 41 cytokines (Ahola-Olli et al., 2017) were associated any COVID-19, and whether liability to COVID-19 affected any of these cytokines. We also assessed whether any findings for any COVID-19 replicated for hospitalized COVID-19.

## Materials and Methods

This is a two-sample bi-directional MR study design, using summary statistics from the latest available genome-wide association study (GWAS) for cytokines and COVID-19. MR is an instrumental variable analysis using genetic instruments, which is less confounded due to randomization of genetic variants at conception. MR relies on three assumptions, i.e., the genetic instruments are associated with the exposure (relevance), the genetic instruments are not linked with confounders of exposure on outcome (independence), and the genetic instruments are linked to the outcome only via the exposure (exclusion-restriction) (Davies et al., 2018).

### Data Sources

#### Genetic Associations With Cytokines

Genetic associations with 41 plasma/serum cytokines (in standard deviations) were obtained from a meta-analysis of up to 8,293 individuals from two studies in Finland, the Cardiovascular Risk in Young Finns Study (*n* = 2,019, mean age 37.4 years in men, 37.5 in women) and the FINRISK survey (*n* = 6,313, survey 1997: mean age 48.3 years in men, 47.3 in women; survey 2002: 60.4 in men, 60.1 in women) (Ahola-Olli et al., 2017). Genetic associations were adjusted for age, sex, body mass index and the first 10 genetic principal components, and used genomic control to correct for population stratification and cryptic relatedness (Ahola-Olli et al., 2017).

#### Genetic Associations With COVID-19

Genetic associations with any COVID-19 compared to the population (36,590 cases and 1,668,938 controls) were obtained from the COVID19-hg GWAS meta-analyses round five summary statistics (updated January 2021, leaving out 23andMe) initiated by the COVID-19 Host Genetics Initiative (COVID-19 Host Genetics Initiative, 2020). Any COVID-19 was defined as laboratory confirmation of SARS-CoV-2 infection (RNA and/or serology based), EHR/ICD coding/physician confirmed COVID-19, or self-reported COVID-19 positive (36,590 cases compared to 1,668,938 population controls). Genetic associations with hospitalized COVID-19 (hospitalized laboratory confirmed) (12,888 cases) compared to the population (1,295,966 controls) were obtained from the same study. Summary statistics leaving out the UK Biobank [any COVID-19 (42,557 cases and 1,424,707 controls), hospitalized COVID-19 (11,829 cases and 1,725,210 controls)] were used as sensitivity analyses as the study had fewer genetic variants. The participants are largely drawn from on-going cohort studies (Supplementary Table 1), mostly from Europe and the United States. The analyses controlled for age, sex, and ancestry (COVID-19 Host Genetics Initiative, 2020).

### Mendelian Randomization Analysis

#### Selection of Genetic Predictors

We used single-nucleotide polymorphisms (SNPs) strongly (*P* < 5 × 10^–8^) and independently (*r*^2^ < 0.05) associated with the exposures (cytokine or the COVID-19 phenotype) as genetic predictors. Given such genetic predictors were not available for all exposures, we also used a less stringent selection criteria (*P* < 5 × 10^–6^) for exposures without predictors at genome-wide significance. Potentially palindromic SNPs (allele pairs A/T or G/C) were removed before SNP selection because allele frequency is not available for the cytokine GWAS precluding unequivocal allele alignment across both studies. Independent genetic predictors (*r*^2^ < 0.05) available for both cytokines and COVID-19 were used in the MR analysis.

#### Statistical Analysis

The *F*-statistic was used as an indicator of instrument strength, obtained by averaging the SNP specific *F*-statistics (square of beta for exposure divided by its variance) (Bowden et al., 2016b). To test independence, we checked whether any of the genetic instruments were associated with several common potential confounders including tobacco smoking status, frequency of alcohol intake and frequency of walking at genome-wide significance, using UK Biobank genetic summary associations^10^.

Estimates were obtained by meta-analyzing SNP-specific Wald estimates (genetic association with outcome divided by association with exposure) using inverse variance weighting (IVW) with fixed (≤3 SNPs) or multiplicative random effects (4 + SNPs), which assumes balanced pleiotropy (Burgess et al., 2013). Given we assessed associations primarily between cytokines and any COVID-19 and the two COVID-19 phenotypes are correlated, we used a Benjamini-Hochberg false discovery rate (FDR)-control to correct for multiple hypotheses testing for 41 cytokines versus each COVID-19 phenotype (Benjamini and Hochberg, 1995).

#### Sensitivity Analysis

MR-Egger was used to identify potentially invalid estimates from IVW (indicated by a non-zero intercept), although it has less statistical power (Bowden et al., 2015). Weighted median (WM) gives robust estimates even when some invalid instruments (<50% weight) are used (Bowden et al., 2016a).

Statistical analyses were conducted using R version 3.6.2 (The R Foundation for Statistical Computing, Vienna, Austria). The “ld_clump” function (“ieugwasr” package) was used to select the independent genetic variants. The MendelianRandomization R package was used to obtain MR estimates. This study only uses published or publicly available data. Ethical approval for each of the studies included in the investigation can be found in the original publications (including informed consent from each participant).

## Results

### Genetic Predictors for Circulating Cytokines and COVID-19

Of the 41 circulating cytokines 19 had genome-wide significant predictors for any COVID-19 (Supplementary Table 2), all the 41 cytokines considered had 2 or more non-palindromic independent genetic predictors with *p*-values smaller than 5 × 10^–6^. The *F*-statistics ranged from 18.7 to 169.8 (Supplementary Table 2). Both COVID-19 phenotypes considered had 2 or more non-palindromic independent genetic predictors with *p*-value < 5 × 10^–8^ (Supplementary Table 3). *F*-statistics ranged from 60.5 to 64.8 (Supplementary Table 3).

### Genetically Predicted Circulating Cytokines on Any COVID-19

**FIGURE 1:**
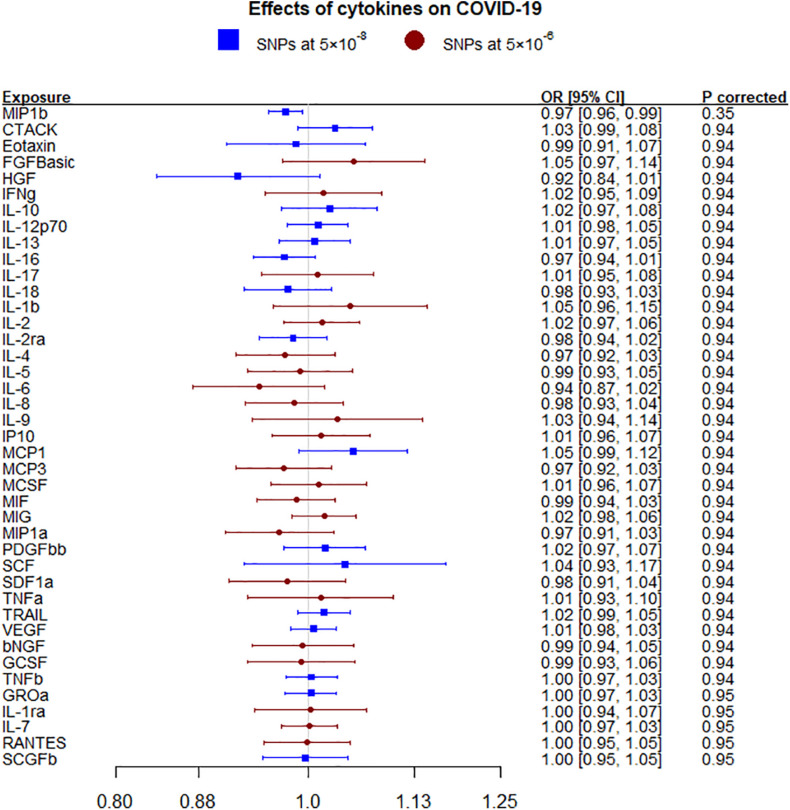
Effects of 41 circulating cytokines on COVID-19 using inverse variance weighting with *p*-values using a Benjamini-Hochberg correction.

Macrophage inflammatory protein-1β (MIP1b) was nominally inversely associated with any COVID-19 using IVW and WM but not after correcting for multiple comparisons (Figure 1 and Supplementary Table 4). These nominal associations replicated for hospitalized COVID-19 using all methods but not after correcting for multiple comparisons (Supplementary Figure 1 and Supplementary Table 5). MIP1b was also found associated with very severe respiratory confirmed COVID-19 (death or respiratory support, 5,582 cases versus 7,09,010 controls) (OR 0.92, 95% CI 0.84–1.00). The other cytokines considered, including IL-6, were not associated with either COVID-19 phenotype (any or hospitalized). In sensitivity analysis using genetic associations leaving out the UK Biobank rather than 23andMe, monocyte chemotactic protein-1 (MCP1; CCL2) was nominally positively associated with any COVID-19 (Supplementary Table 6) and MIP1b was also nominally inversely associated with hospitalized COVID-19 (Supplementary Table 7).

### Genetically Predicted Liability to COVID-19 on Circulating Cytokines

**FIGURE 2:**
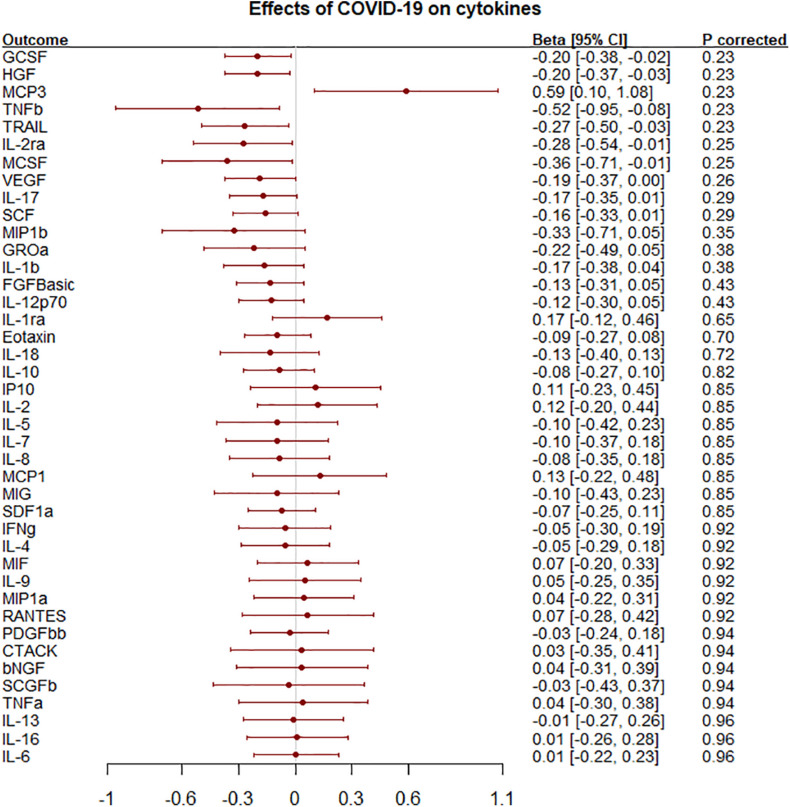
Effects of liability to COVID-19 on 41 circulating cytokines using inverse variance weighting with *p-*values using a Benjamini-Hochberg correction.

Liability to any COVID-19 was nominally inversely associated with granulocyte colony-stimulating factor (GCSF), hepatocyte growth factor (HGF), IL-2 receptor alpha subunit (IL2ra), macrophage colony-stimulating factor (MCSF), tumor necrosis factor-beta (TNFb) and TNF-related apoptosis inducing ligand (TRAIL) but positively associated with monocyte chemotactic protein-3 (MCP3) but not after correction for multiple comparisons (Figure 2 and Supplementary Table 8). The association of liability to hospitalized COVID-19 with IL2ra and TNFb were similar to those for liability to any COVID-19 (Supplementary Figure 2 and Supplementary Table 9). Most results were similar using genetic associations leaving out the UK Biobank rather than 23andMe (Supplementary Tables 10, 11).

## Discussion

In this bi-directional MR study of circulating cytokines and COVID-19, we found some associations in both directions. For associations of cytokines with COVID-19, lower MIP1b was nominally and consistently associated with COVID-19 phenotypes, which has rarely been studied previously. In the other direction, we found liability to COVID-19 was inversely associated with several cytokines, such as GCSF and HGF.

Few previous studies have investigated the role of MIP1b in COVID-19, although higher MIP1b in patients than controls has been observed (De Biasi et al., 2020; Hachim et al., 2020). This study showed internally consistent associations of MIP1b with COVID-19, indicating MIP1b may play a protective role in both COVID-19 vulnerability and hospitalization. MIP1b is a chemoattractant for several immune cells, particularly T cells (Schall et al., 1993; Tedla et al., 1998; Bystry et al., 2001), whose function is increasingly being recognized in COVID-19 (Chen and John Wherry, 2020). T cells are pivotal to the anti-viral immune response, and may be low and/or functionally exhausted in COVID-19 (Mathew et al., 2020) especially severe COVID-19 (Diao et al., 2020; Giamarellos-Bourboulis et al., 2020). In this study, we also found liability to COVID-19 might reduce MIP1b levels using MR-Egger but not any other methods, whether it is relevant in the T cell exhaustion in COVID-19 needs further investigation. Given the cytokines were obtained from studies conducted before the COVID-19 pandemic, any associations of liability to COVID-19 with cytokines indicate that people more vulnerable to COVID-19 have a somewhat different cytokine profile possibly reflecting differences in immune functioning.

Although the evidence concerning the role of MIP1b in COVID-19 is inevitably limited, MIP1b has been extensively investigated in other viral diseases, such as HIV (Cocchi et al., 2000) and hepatitis A (Sung et al., 2017). MIP1b is a ligand of CCR5, which has also been extensively investigated in the context of HIV (Qi et al., 2020). Relevance of IL-6 to specifically COVID-19 has been more intensively investigated. Observationally associations of IL-6 with COVID-19 have been seen (Chen et al., 2020; Gao et al., 2020; Ruan et al., 2020). Genetically mimicking IL6R blockade using genetic instruments from the *IL6R* gene strongly associated with c-reactive protein was associated with lower risk of hospitalized COVID-19 (Bovijn et al., 2020), but whether these associations are due to IL-6, IL-6R, or pleiotropic effects via other mechanisms is unclear. Genetically mimicking IL6R blockers, such as tocilizumab, suggest a very wide range of effects (Rosa et al., 2019).

MR studies rely on three important assumptions, i.e., relevance, independence and exclusion restriction. We used several methods to detect violation of these assumptions. First, to ensure relevance we selected genetic predictors strongly (genome-wide significant) associated with the exposures, but also included less significant predictors 5 × 10^–6^ for some exposures because predictors at genome-wide significance were not available for all. The *F*-statistics did not indicate weak instruments. Second, to check independence, we assessed associations of the genetic predictors with several common confounders, and found no associations with these confounders (Supplementary Tables 12–17), except one SNP rs3748034 predicting HGF was associated with alcohol intake. Third, to test the exclusion restriction assumption, we used sensitivity analyses with different assumptions including MR-Egger and WM to detect pleiotropy and give robust estimates even if invalid SNPs are included, but might lack of power (such as MR-Egger).

This large bi-directional MR study used the largest available GWASs for cytokines and COVID-19. However, limitations still exist. First, we assumed genetic associations in one sample could be replicated in the other sample (Burgess et al., 2015). Both GWAS mainly concern people of European ancestry. Second, we assumed linear associations in this study, while the associations of cytokines and COVID-19 could be complex and change over time. Third, given most participants were from Europe and the United States, whether the associations are applicable to other populations, such as Asians, is uncertain. However, causal factors should be consistent across different populations although the underlying mechanism might not always be relevant^11^. Fourth, covariable adjusted summary statistics might bias the MR results in some situations (Hartwig et al., 2019). Here, the summary statistics for cytokines have adjusted for body mass index, which is the common cause of COVID-19 (Mark et al., 2020) and survival (Sun et al., 2019). As such, the adjustment is more likely to correct for selection bias more than induce it (Schooling et al., 2020). Fifth, the case definition is essentially nested meaning the phenotypes are not completely independent. We focused on any COVID-19 to maximize power and comprehensiveness, particularly given COVID-19 can have long-term consequences even if the initial disease is not severe^12^. Sixth, the case definition for any COVID-19 reflects disease rather than infection with SARS-CoV-2, so our findings may represent the role of MIP1b in preventing disease on infection or in protecting against infection. Seventh, there were suggestive associations of liability to COVID-19 with MIP1b using MR-Egger. We used Steiger filtering to indicate whether there were any invalid genetic instruments that predicted the outcomes more than the exposures by comparing the variance in exposures and outcomes explained by each genetic variant (*r*^2^). For the associations of MIP1b with COVID-19, Steiger filtering did not indicate any invalid genetic instruments. However, Steiger filtering did suggest two genetic instruments might be predictors of MIP1b more than of COVID-19. After removing them, the observed association of liability to COVID-19 with MIP1b was not evident using any method (Supplementary Table 18). Last, but not least, we did not consider the relations among cytokines and the role of the cytokine cascade in immune response.

## Conclusion

This large bi-directional MR study suggests MIP1b may protect against any COVID-19 and also hospitalized COVID-19. MIP1b has previously been investigated as a target in other infectious diseases, whether it could be a potential target for prevention and treatment of COVID-19 as well as its exact role in immune response needs further investigation.

## Data Availability Statement

The datasets generated and/or analysed during the current study are available in Supplementary Files.

## Ethics Statement

This study only uses published or publicly available data. Ethical approval for each of the studies included in the investigation can be found in the original publications (including informed consent from each participant).

## Author Contributions

CS and ML designed the study. ML conducted the analysis and drafted the manuscript. CS directed the analytic strategy and supervised the study from conception to completion. CY checked the data analyses. ML, CY, and CS revised the drafts of the manuscript. All authors contributed to the interpretation of the data and critically revising the manuscript.

## Conflict of Interest

The authors declare that the research was conducted in the absence of any commercial or financial relationships that could be construed as a potential conflict of interest.

## Footnotes

1 https://www.who.int/emergencies/diseases/novel-coronavirus-2019/situation-reports

2 https://www.nidirect.gov.uk/articles/coronavirus-covid-19-definitions-clinically-extremely-vulnerable-and-vulnerable

3 https://clinicaltrials.gov/ct2/show/NCT04443881

4 https://www.reuters.com/article/us-health-coronavirus-roche-hldg/roche-rheumatoid-arthritis-drug-fails-to-help-covid-19-patients-in-italian-study-idUSKBN23O3GG

5 https://pharmaphorum.com/news/il-6-hypothesis-looking-shaky-as-kevzara-fails-covid-19-trial/

6 https://pharmaphorum.com/news/jury-still-out-on-il-6-drugs-in-covid-19-following-preliminary-trial-results/

7 https://www.roche.com/investors/updates/inv-update-2020-07-29.htm

8 https://www.roche.com/investors/updates/inv-update-2020-09-18.htm

9 https://pubmed.ncbi.nlm.nih.gov/28569269/

10 http://www.nealelab.is/uk-biobank/

11 https://pubmed.ncbi.nlm.nih.gov/31121303/

12 https://covid.joinzoe.com/post/long-covid
