# Supplementary material for: Circulating Cytokines and Coronavirus Disease: A Bi-Directional Mendelian Randomization Study
Source: Front Genet. 2021 Jun 7;12:680646. doi: 10.3389/fgene.2021.680646 (PMC8215612; doi:10.3389/fgene.2021.680646)
Supplement: Supplementary file 1 [file Data_Sheet_1.pdf]

```
library(MendelianRandomization)
library(readr)
library(metafor)
library(data.table)
library(dplyr)
library(plyr)
library(devtools)
library(TwoSampleMR)
library(tidyverse)
require(ggplot2)

##extract independent genetic predictors of three covid phenotypes removing palindromic snps
#1 extract snps at 5*10-8 predicting covid
#2 give them rsid
#3 get independent snps using clumping
var<-fread("variants.tsv")[,c("rsid","chr","pos")]
var$chrpos<-paste(var$chr,":",var$pos,sep="")
#exp1:hospitalized covid vs population
out<-fread("COVID19_HGI_B2_ALL_leave_23andme_20210107.b37.txt.gz")
exp<-out[out$all_inv_var_meta_p < 0.00000005,]
rm(out)
exp$allele<-paste(exp$REF,exp$ALT,sep="")
exp<-exp[exp$allele!="AT",]
exp<-exp[exp$allele!="TA",]
exp<-exp[exp$allele!="CG",]
exp<-exp[exp$allele!="GC",]
exp$chrpos<-paste(exp$`#CHR`,":",exp$POS,sep="")
exp<-merge(exp,var, by="chrpos",all.x=TRUE)
exp$pval<-exp$all_inv_var_meta_p
exp$rsid<-ifelse(is.na(exp$rsid.y),exp$rsid.x,exp$rsid.y)
exp_dat<-ieugwasr::ld_clump(exp,clump_r2 = 0.05)
```

```
write.csv(exp_dat,file=" hospitalized covid exposure dataset after clumping 8.csv",row.names=FALSE)


#exp2:covid vs population
memory.limit(200500)
out<-fread("COVID19_HGI_C2_ALL_leave_23andme_20210107.b37.txt.gz",header = T)
exp<-out[out$all_inv_var_meta_p < 0.00000005,]
rm(out)
exp$allele<-paste(exp$REF,exp$ALT,sep="")
exp<-exp[exp$allele!="AT",]
exp<-exp[exp$allele!="TA",]
exp<-exp[exp$allele!="CG",]
exp<-exp[exp$allele!="GC",]
exp$chrpos<-paste(exp$`#CHR`,":",exp$POS,sep="")
exp<-merge(exp,var, by="chrpos",all.x=TRUE)
exp$pval<-exp$all_inv_var_meta_p
exp$rsid<-ifelse(is.na(exp$rsid.y),exp$rsid.x,exp$rsid.y)
exp_dat<-ieugwasr::ld_clump(exp,clump_r2 = 0.05)
write.csv(exp_dat,file="covid exposure dataset after clumping 8.csv",row.names=FALSE)
rm(var)


##the function to extract MR results
foo <- function(MRivwEs,MREggerEs,MRWMs){
  res<-NA
  res<-as.data.frame(res)
  res$beta_IVW<-MRivwEs$Estimate
  res$CIL_IVW<-MRivwEs$CILower
  res$CIU_IVW<-MRivwEs$CIUpper
  res$CI_IVW<- paste(round(MRivwEs$CILower, digit=2),", ",round(MRivwEs$CIUpper, digit=2), sep = "")
  res$p_IVW<-MRivwEs$Pvalue
  res$nsnp<-MRivwEs$SNPs
```

```r
  if (length((MREggerEs))==0)
  {return(res)}
  else{
    res$beta_EGG<-MREggerEs$Estimate
    res$CIL_EGG<-MREggerEs$CILower.Est
    res$CIU_EGG<-MREggerEs$CIUpper.Est
    res$CI_EGG<- paste(round(MREggerEs$CILower.Est, digit=2),", ",round(MREggerEs$CIUpper.Est, 
digit=2), sep = "")
    res$p_EGG<-MREggerEs$Pvalue.Est
    res$p_int_EGG<-MREggerEs$Pvalue.Int
    res$i2_EGG<-MREggerEs$I.sq
    res$beta_WM<-MRWMs$Estimate
    res$CIL_WM<-MRWMs$CILower
    res$CIU_WM<-MRWMs$CIUpper
    res$CI_WM<- paste(round(MRWMs$CILower, digit=2),", ",round(MRWMs$CIUpper, digit=2), sep = 
"")
    res$p_WM<-MRWMs$Pvalue
    return(res)
  }}

tar_list<-fread("target_list.csv",header = T )
for (i in (1:length(tar_list$exposure)))
{
  filename_out<-paste(tar_list[i,1],".data.gz",sep="")
  out<-read.table(filename_out,header=TRUE)
  for (j in c(1:3))

  { filename_exp<-paste(tar_list[j,3]," exposure dataset after clumping 8.csv",sep="")
  exp<-fread(filename_exp,header=TRUE)
  outcome<-out[out$MarkerName %in% exp$rsid,]
  outcome$OtherAllele<-toupper(outcome$OtherAllele)
  outcome$EffectAllele<-toupper(outcome$EffectAllele)
```

```
  exp_dat<- format_data(exp,type = "exposure",

snp_col="rsid",beta_col="all_inv_var_meta_beta",se_col="all_inv_var_meta_sebeta",eaf_col="eaf",
                  effect_allele_col="ALT",other_allele_col="REF")
  outcome_dat<- format_data(outcome,type = "outcome",
                     snp_col="MarkerName",beta_col="Effect",se_col="StdErr",
                     effect_allele_col="EffectAllele",other_allele_col="OtherAllele",
                     pval_col="P.value")


  dat_harmo<-harmonise_data(exposure_dat = exp_dat,
                     outcome_dat = outcome_dat,
                     action = 2)
  filename<-paste(tar_list[j,3]," on ",tar_list[i,1]," harmonized dataset removing palindromic
snp.csv",sep="")
  write.csv(dat_harmo, filename, row.names = F)
  dat_harmo<-dat_harmo[dat_harmo$mr_keep==TRUE,]
  MRinputeobject <- MendelianRandomization::mr_input(bx=dat_harmo$beta.exposure,
                                    by=dat_harmo$beta.outcome,
                                    bxse=dat_harmo$se.exposure,
                                    byse=dat_harmo$se.outcome)
  MRivwEs <- MendelianRandomization::mr_ivw(MRinputeobject)
  MRivwEs
  MREggerEs <- MendelianRandomization::mr_egger(MRinputeobject)
  MREggerEs
  MRWMs<- MendelianRandomization::mr_median(MRinputeobject)
  MRWMs
  result<-foo(MRivwEs=MRivwEs,MREggerEs=MREggerEs,MRWMs=MRWMs)
  filename1<-paste(tar_list[j,3]," on ",tar_list[i,1]," mr results removing palindromic snp.csv",sep="")
  write.csv(result,file=filename1,row.names=F)
  filename2<-paste(tar_list[j,3]," on ",tar_list[i,1]," mr results removing palindromic snp.txt",sep="")
  capture.output(MRivwEs, MREggerEs, MRWMs, file = filename2, split = FALSE)
  }}
```

```
###combine results into a document -8
tar_list<-fread(target_list.csv",header = T )
res<-data.frame()
for (i in (1:length(tar_list$exposure)))
{  for (j in c(2:2))
{
  filename<-paste(tar_list[j,3]," on ",tar_list[i,1]," mr results removing palindromic snp.csv",sep="")
  res1<-read.csv(file=filename, header = TRUE)
  res1$association<-paste(tar_list[j,3]," on ",tar_list[i,1])
  res<-dplyr::bind_rows(res,res1)
  res
}}
res$exp<-tar_list$exposure
head(res)
#BH method to correct for multiple testing
res$p_adj<-p.adjust(res$p_IVW, method = "BH", n = length(res$p_IVW))
write.csv(res, file="hospitalized covid on cytokine mr results using snps at 8 updated 2021
jan.csv",row.names = F )


res<-data.frame()
for (i in (1:length(tar_list$exposure)))
{  for (j in c(3:3))
{
  filename<-paste(tar_list[j,3]," on ",tar_list[i,1]," mr results removing palindromic snp.csv",sep="")
  res1<-read.csv(file=filename, header = TRUE)
  res1$association<-paste(tar_list[j,3]," on ",tar_list[i,1])
  res<-dplyr::bind_rows(res,res1)
  res
}}
head(res)
```

```
res$exp<-tar_list$exposure

#BH method to correct for multiple testing

res$p_adj<-p.adjust(res$p_IVW, method = "BH", n = length(res$p_IVW))

write.csv(res, file="covid on cytokine mr results using snps at 8 updated 2021 jan.csv",row.names =
F )


###calculate f statistics

tar_list<-fread("target_list.csv",header = T )


for (j in (1:3))

{

  filename_exp<-paste(tar_list[j,3]," exposure dataset after clumping 8.csv",sep="")

  exp<-fread(filename_exp,header=TRUE)

  exp$Fsta<-exp$all_inv_var_meta_beta^2/exp$all_inv_var_meta_sebeta^2

  write.csv(exp, file = filename_exp, row.names = F)

}


tar_list<-fread("target_list.csv",header = T )

res_fsta<-data.frame()

for (j in (1:3))

{

  filename_exp<-paste(tar_list[j,3]," exposure dataset after clumping 8.csv",sep="")

  exp<-fread(filename_exp)

  fsta<-mean(exp$Fsta)

  fsta<-as.data.frame(fsta)

  res_fsta<-rbind(res_fsta,fsta)

}

res_fsta$exposure<-tar_list$outcome[c(1:3)]

write.csv(res_fsta,file= "F statistics for SNPs at 10-8.csv",row.names = F)


###combine harmonized dataset
```

```
#covid on cytokine

sort<-fread("C:/Transfer/D drive/cytokine and covid/target_list.csv",header = T )
a<-c("hospitalized covid")
res<-data.frame()
for (i in (1:length(sort$exposure)))
{ for (j in (1:length(a)))
{
  filename<-paste(a[j]," on ",sort[i,1], " harmonized dataset removing palindromic snp.csv",sep="")
  res1<-read.csv(file=filename, header = TRUE)
  res1$outcome<-as.character(sort[i,1])
  res1$exposure<-"hospitalized covid"
  res<-rbind(res,res1)
  res
}}
res<-res[,c("SNP","effect_allele.exposure" ,"other_allele.exposure" , "effect_allele.outcome",
"other_allele.outcome" ,  "beta.exposure","se.exposure","beta.outcome" , "se.outcome" ,
"pval.exposure" ,"exposure","outcome")]
write.csv(res, file="hospitalized covid on cytokine summary statistics updated 2021
jan.csv",row.names = F )

sort<-fread("C:/Transfer/D drive/cytokine and covid/target_list.csv",header = T )
a<-c("covid")
res<-data.frame()
for (i in (1:length(sort$exposure)))
{ for (j in (1:length(a)))
{
  filename<-paste(a[j]," on ",sort[i,1], " harmonized dataset removing palindromic snp.csv",sep="")
  res1<-read.csv(file=filename, header = TRUE)
  res1$outcome<-as.character(sort[i,1])
  res1$exposure<-"covid"
  res<-rbind(res,res1)
```

```
  res
}}
res<-res[,c("SNP","effect_allele.exposure" ,"other_allele.exposure" , "effect_allele.outcome",
"other_allele.outcome" ,  "beta.exposure","se.exposure","beta.outcome" , "se.outcome" ,
"pval.exposure" ,"exposure","outcome")]
write.csv(res, file="covid on cytokine summary statistics updated 2021 jan.csv",row.names = F )
```
