# Supplementary material for: Circulating Cytokines and Coronavirus Disease: A Bi-Directional Mendelian Randomization Study
Source: Front Genet. 2021 Jun 7;12:680646. doi: 10.3389/fgene.2021.680646 (PMC8215612; doi:10.3389/fgene.2021.680646)
Supplement: Supplementary file 2 [file Data_Sheet_2.pdf]

```
library(MendelianRandomization)

library(readr)

library(metafor)

library(data.table)

library(dplyr)

library(plyr)

library(devtools)

library(TwoSampleMR)

library(tidyverse)

require(ggplot2)


#extract uncorrelated genetic variants

tar_list<-fread("target_list.csv",header = T ) #list of cytokines

for (i in (1:length(tar_list$exposure)))

{

  filename<-paste(tar_list[i,1],".data.gz",sep="")

  exp<-read.table(filename,header = T)

  exp$allele<-paste(exp$EffectAllele,exp$OtherAllele,sep="")

  exp<-exp[exp$allele!="at",]

  exp<-exp[exp$allele!="ta",]

  exp<-exp[exp$allele!="cg",]

  exp<-exp[exp$allele!="gc",]

  exp<-exp[exp$'P.value' < 0.000005,]

  exp$rsid<-exp$MarkerName

  exp$pval<-exp$P.value

  exp<- ieugwasr::ld_clump(exp,clump_r2 = 0.05)

  exp$chrpos<-paste(exp$Chromosome,":",exp$Position,sep="")

  filename1<-paste(tar_list[i,1], " exposure dataset removing palindromic snp after clumping.csv",sep="")

  write.csv(exp,filename1, row.names = F )

}
```

```
##the function to extract MR results
foo <- function(MRivwEs,MREggerEs,MRWMs){
  res<-NA
  res<-as.data.frame(res)
  res$OR_IVW<-exp(MRivwEs$Estimate)
  res$CIL_IVW<-exp(MRivwEs$CILower)
  res$CIU_IVW<-exp(MRivwEs$CIUpper)
  res$CI_IVW<- paste(round(exp(MRivwEs$CILower), digit=2),", ",round(exp(MRivwEs$CIUpper), 
digit=2), sep = "")
  res$p_IVW<-MRivwEs$Pvalue
  res$nsnp<-MRivwEs$SNPs
  res$p_hetero<-MRivwEs$Heter.Stat[2]
  if (length((MREggerEs))==0)
  {return(res)}
  else{
    res$OR_EGG<-exp(MREggerEs$Estimate)
    res$CIL_EGG<-exp(MREggerEs$CILower.Est)
    res$CIU_EGG<-exp(MREggerEs$CIUpper.Est)
    res$CI_EGG<- paste(round(exp(MREggerEs$CILower.Est), digit=2),", 
",round(exp(MREggerEs$CIUpper.Est), digit=2), sep = "")
    res$p_EGG<-MREggerEs$Pvalue.Est
    res$p_int_EGG<-MREggerEs$Pvalue.Int
    res$i2_EGG<-MREggerEs$I.sq
    res$OR_WM<-exp(MRWMs$Estimate)
    res$CIL_WM<-exp(MRWMs$CILower)
    res$CIU_WM<-exp(MRWMs$CIUpper)
    res$CI_WM<- paste(round(exp(MRWMs$CILower), digit=2),", ",round(exp(MRWMs$CIUpper), 
digit=2), sep = "")
    res$p_WM<-MRWMs$Pvalue
    return(res)
  }}
memory.limit(200500)
```

```
tar_list<-fread("target_list.csv",header = T )

###run MR analysis

##outcome1:hospitalized covid vs population

out<-fread("COVID19_HGI_B2_ALL_leave_23andme_20210107.b37.txt.gz")

out$chrpos<-paste(out$`#CHR`,":",out$POS,sep="")

for (i in (1:length(tar_list$exposure)))

{

  filename_exp<-paste(tar_list[i,1], " exposure dataset removing palindromic snp after
clumping.csv",sep="")

  exp<-fread(filename_exp)

  exp$EffectAllele<-toupper(exp$EffectAllele)

  exp$OtherAllele<-toupper(exp$OtherAllele)

  outcome<-merge(out, exp, by.x="chrpos",by.y ="chrpos")

  exp_dat<- format_data(exp,type = "exposure",

                    snp_col="MarkerName",beta_col="Effect",se_col="StdErr",

                    effect_allele_col="EffectAllele",other_allele_col="OtherAllele",

                    pval="pval")

  outcome_dat<- format_data(outcome,type = "outcome",
snp_col="rsid.y",beta_col="all_inv_var_meta_beta",se_col="all_inv_var_meta_sebeta",eaf_col="all_
meta_AF", effect_allele_col="ALT",other_allele_col="REF")

  dat_harmo<-harmonise_data(exposure_dat = exp_dat,

                      outcome_dat = outcome_dat,

                      action = 2)

  filename<-paste(tar_list[i,1]," on hospitalized covid vs population harmonized dataset removing
palindromic snp.csv",sep="")

  write.csv(dat_harmo, filename, row.names = F)

  dat_harmo<-dat_harmo[dat_harmo$mr_keep==TRUE,]

  MRinputeobject <- MendelianRandomization::mr_input(bx=dat_harmo$beta.exposure,

                                         by=dat_harmo$beta.outcome,

                                         bxse=dat_harmo$se.exposure,

                                         byse=dat_harmo$se.outcome)

  MRivwEs <- MendelianRandomization::mr_ivw(MRinputeobject)
```

```
  MRivwEs
  MREggerEs <- MendelianRandomization::mr_egger(MRinputeobject)
  MREggerEs
  MRWMs<- MendelianRandomization::mr_median(MRinputeobject)
  MRWMs
  result<-foo(MRivwEs=MRivwEs,MREggerEs=MREggerEs,MRWMs=MRWMs)
  filename1<-paste(tar_list[i,1]," on hospitalized covid vs population mr results removing palindromic snp.csv",sep="")
  write.csv(result,file=filename1,row.names=F)
  filename2<-paste(tar_list[i,1]," on hospitalized covid vs population mr results removing palindromic snp.txt",sep="")
  capture.output(MRivwEs, MREggerEs, MRWMs, file = filename2, split = FALSE)
}
rm(out)

##outcome2:covid vs population
memory.limit(201500)
out<-fread("COVID19_HGI_C2_ALL_leave_23andme_20210107.b37.txt.gz",header = T)
out$chrpos<-paste(out$`#CHR`,":",out$POS,sep="")
for (i in (1:length(tar_list$exposure)))
{
  filename_exp<-paste(tar_list[i,1], " exposure dataset removing palindromic snp after clumping.csv",sep="")
  exp<-fread(filename_exp)
  exp$EffectAllele<-toupper(exp$EffectAllele)
  exp$OtherAllele<-toupper(exp$OtherAllele)
  outcome<-merge(out, exp, by.x="chrpos",by.y ="chrpos")
  exp_dat<- format_data(exp,type = "exposure",
             snp_col="MarkerName",beta_col="Effect",se_col="StdErr",
             effect_allele_col="EffectAllele",other_allele_col="OtherAllele",
             pval_col="pval")
  outcome_dat<- format_data(outcome,type = "outcome",
```

```
snp_col="rsid.y",beta_col="all_inv_var_meta_beta",se_col="all_inv_var_meta_sebeta",
                        effect_allele_col="ALT",other_allele_col="REF",eaf_col="all_meta_AF")
  dat_harmo<-harmonise_data(exposure_dat = exp_dat,
                        outcome_dat = outcome_dat,
                        action = 2)
  filename<-paste(tar_list[i,1]," on covid vs population harmonized dataset removing palindromic snp.csv",sep="")
  write.csv(dat_harmo, filename, row.names = F)
  dat_harmo<-dat_harmo[dat_harmo$mr_keep==TRUE,]
  MRinputeobject <- MendelianRandomization::mr_input(bx=dat_harmo$beta.exposure,
                                             by=dat_harmo$beta.outcome,
                                             bxse=dat_harmo$se.exposure,
                                             byse=dat_harmo$se.outcome)
  MRivwEs <- MendelianRandomization::mr_ivw(MRinputeobject)
  MRivwEs
  MREggerEs <- MendelianRandomization::mr_egger(MRinputeobject)
  MREggerEs
  MRWMs<- MendelianRandomization::mr_median(MRinputeobject)
  MRWMs
  result<-foo(MRivwEs=MRivwEs,MREggerEs=MREggerEs,MRWMs=MRWMs)
  filename1<-paste(tar_list[i,1]," on covid vs population mr results removing palindromic snp.csv",sep="")
  write.csv(result,file=filename1,row.names=F)
  filename2<-paste(tar_list[i,1]," on covid vs population mr results removing palindromic snp.txt",sep="")
  capture.output(MRivwEs, MREggerEs, MRWMs, file = filename2, split = FALSE)
}
rm(list=ls(all=TRUE))


###analysis usng snps at genome wide significance
tar_list<-fread("target_list.csv",header = T )
```

```
foo <- function(MRivwEs,MREggerEs,MRWMs){
  res<-NA
  res<-as.data.frame(res)
  res$OR_IVW<-exp(MRivwEs$Estimate)
  res$CIL_IVW<-exp(MRivwEs$CILower)
  res$CIU_IVW<-exp(MRivwEs$CIUpper)
  res$CI_IVW<- paste(round(exp(MRivwEs$CILower), digit=2),", ",round(exp(MRivwEs$CIUpper), digit=2), sep = "")
  res$p_IVW<-MRivwEs$Pvalue
  res$nsnp<-MRivwEs$SNPs
  res$p_hetero<-MRivwEs$Heter.Stat[2]
  if (length((MREggerEs))==0)
  {return(res)}
  else{
    res$OR_EGG<-exp(MREggerEs$Estimate)
    res$CIL_EGG<-exp(MREggerEs$CILower.Est)
    res$CIU_EGG<-exp(MREggerEs$CIUpper.Est)
    res$CI_EGG<- paste(round(exp(MREggerEs$CILower.Est), digit=2),", ",round(exp(MREggerEs$CIUpper.Est), digit=2), sep = "")
    res$p_EGG<-MREggerEs$Pvalue.Est
    res$p_int_EGG<-MREggerEs$Pvalue.Int
    res$i2_EGG<-MREggerEs$I.sq
    res$OR_WM<-exp(MRWMs$Estimate)
    res$CIL_WM<-exp(MRWMs$CILower)
    res$CIU_WM<-exp(MRWMs$CIUpper)
    res$CI_WM<- paste(round(exp(MRWMs$CILower), digit=2),", ",round(exp(MRWMs$CIUpper), digit=2), sep = "")
    res$p_WM<-MRWMs$Pvalue
    return(res)
  }}
a<-c("hospitalized covid","covid")
for (i in (1:length(tar_list$exposure)))
```

```
{ for (j in (1:length(a)))
{
  filename<-paste(tar_list[i,1]," on ",a[j], " vs population harmonized dataset removing palindromic snp.csv",sep="")
  dat_harmo<-read.csv(file=filename, header = TRUE)
  dat_harmo<-dat_harmo[dat_harmo$pval.exposure<0.00000005,]
  if(nrow(dat_harmo)>1)
  {
    MRinputeobject <- MendelianRandomization::mr_input(bx=dat_harmo$beta.exposure,
                                             by=dat_harmo$beta.outcome,
                                             bxse=dat_harmo$se.exposure,
                                             byse=dat_harmo$se.outcome)
    MRivwEs <- MendelianRandomization::mr_ivw(MRinputeobject)
    MRivwEs
    MREggerEs <- MendelianRandomization::mr_egger(MRinputeobject)
    MREggerEs
    MRWMs<- MendelianRandomization::mr_median(MRinputeobject)
    MRWMs
    result<-foo(MRivwEs=MRivwEs,MREggerEs=MREggerEs,MRWMs=MRWMs)
    filename1<-paste(tar_list[i,1]," on ",a[j], " vs population mr results removing palindromic snp 8.csv",sep="")
    write.csv(result,file=filename1,row.names=F)
    filename2<-paste(tar_list[i,1]," on ",a[j], " vs population mr results removing palindromic snp 8.txt",sep="")
    capture.output(MRivwEs, MREggerEs, MRWMs, file = filename2, split = FALSE)
  }
}}

##combine results on  covid -8
tar_list<-fread("target_list.csv",header = T )
a<-c("hospitalized covid")
res<-data.frame()
```

```
for (i in (1:length(tar_list$exposure)))
{ for (j in (1:length(a)))
{
  filename<-paste(tar_list[i,1]," on ",a[j], " vs population harmonized dataset removing palindromic snp.csv",sep="")
  dat_harmo<-read.csv(file=filename, header = TRUE)
  dat_harmo<-dat_harmo[dat_harmo$pval.exposure<0.00000005,]
  if(nrow(dat_harmo)>1)
  { filename<-paste(tar_list[i,1]," on ",a[j], " vs population mr results removing palindromic snp 8.csv",sep="")
  res1<-read.csv(file=filename, header = TRUE)
  res1$association<-paste(tar_list[i,1]," on ",a[j], " vs population")
  res<-dplyr::bind_rows(res,res1)
  res
  }}}

write.csv(res, file="cytokine on hospitalized covid mr results using snps at 8 updated 2021 jan.csv",row.names = F )

a<-c("covid")
res<-data.frame()
for (i in (1:length(tar_list$exposure)))
{ for (j in (1:length(a)))
{
  filename<-paste(tar_list[i,1]," on ",a[j], " vs population harmonized dataset removing palindromic snp.csv",sep="")
  dat_harmo<-read.csv(file=filename, header = TRUE)
  dat_harmo<-dat_harmo[dat_harmo$pval.exposure<0.00000005,]
  if(nrow(dat_harmo)>1)
  { filename<-paste(tar_list[i,1]," on ",a[j], " vs population mr results removing palindromic snp 8.csv",sep="")
  res1<-read.csv(file=filename, header = TRUE)
  res1$association<-paste(tar_list[i,1]," on ",a[j], " vs population")
```

```
  res<-dplyr::bind_rows(res,res1)
  res
  }}}

write.csv(res, file="cytokine on covid mr results using snps at 8 updated 2021 jan.csv",row.names = F )



##combine results on covid -6
tar_list<-fread("target_list.csv",header = T )
a<-c("hospitalized covid")
res<-data.frame()
for (i in (1:length(tar_list$exposure)))
{ for (j in (1:length(a)))
{
  filename<-paste(tar_list[i,1]," on ",a[j], " vs population mr results removing palindromic snp.csv",sep="")
  res1<-read.csv(file=filename, header = TRUE)
  res1$association<-paste(tar_list[i,1]," on ",a[j], " vs population")
  res<-dplyr::bind_rows(res,res1)
  res
}}
res$exp<-tar_list$exposure
head(res)

write.csv(res, file="cytokine on hospitalized covid mr results using snps at 6 updated 2021 jan.csv",row.names = F )

a<-c("covid")
res<-data.frame()
for (i in (1:length(tar_list$exposure)))
{ for (j in (1:length(a)))
```

```
{
  filename<-paste(tar_list[i,1]," on ",a[j], " vs population mr results removing palindromic snp.csv",sep="")
  res1<-read.csv(file=filename, header = TRUE)
  res1$association<-paste(tar_list[i,1]," on ",a[j], " vs population")
  res<-dplyr::bind_rows(res,res1)
  res
}}
res$exp<-tar_list$exposure
head(res)

write.csv(res, file="cytokine on covid mr results using snps at 6 updated 2021 jan.csv",row.names = F )

##combine MR results
###hospitalized covid
dat8<-read.csv("cytokine on hospitalized covid mr results using snps at 8 updated 2021 jan.csv",header=T)
dat6<-read.csv("cytokine on hospitalized covid mr results using snps at 6 updated 2021 jan.csv",header=T)
dat<-merge(dat8,dat6,by='association',all.y=T)
dat$OR_IVW<-ifelse(is.na(dat$OR_IVW.x),dat$OR_IVW.y,dat$OR_IVW.x)
dat$CI_IVW.y<-as.character(dat$CI_IVW.y)
dat$CI_IVW.x<-as.character(dat$CI_IVW.x)
dat$CI_IVW<-ifelse(is.na(dat$OR_IVW.x),dat$CI_IVW.y,dat$CI_IVW.x)
dat$p_IVW<-ifelse(is.na(dat$OR_IVW.x),dat$p_IVW.y,dat$p_IVW.x)
dat$CIL_IVW<-ifelse(is.na(dat$OR_IVW.x),dat$CIL_IVW.y,dat$CIL_IVW.x)
dat$CIU_IVW<-ifelse(is.na(dat$OR_IVW.x),dat$CIU_IVW.y,dat$CIU_IVW.x)
dat$nsnp<-ifelse(is.na(dat$nsnp.x),dat$nsnp.y,dat$nsnp.x)
dat$p_hetero<-ifelse(is.na(dat$p_hetero.x),dat$p_hetero.y,dat$p_hetero.x)

dat$OR_EGG<-ifelse(is.na(dat$OR_IVW.x),dat$OR_EGG.y,dat$OR_EGG.x)
```

```
dat$CIL_EGG<-ifelse(is.na(dat$OR_IVW.x),dat$CIL_EGG.y,dat$CIL_EGG.x)
dat$CIU_EGG<-ifelse(is.na(dat$OR_IVW.x),dat$CIU_EGG.y,dat$CIU_EGG.x)
dat$CI_EGG.y<-as.character(dat$CI_EGG.y)
dat$CI_EGG.x<-as.character(dat$CI_EGG.x)
dat$CI_EGG<-ifelse(is.na(dat$OR_IVW.x),dat$CI_EGG.y,dat$CI_EGG.x)
dat$p_EGG<-ifelse(is.na(dat$OR_IVW.x),dat$p_EGG.y,dat$p_EGG.x)
dat$p_int_EGG<-ifelse(is.na(dat$OR_IVW.x),dat$p_int_EGG.y,dat$p_int_EGG.x)

dat$OR_WM<-ifelse(is.na(dat$OR_IVW.x),dat$OR_WM.y,dat$OR_WM.x)
dat$CIL_WM<-ifelse(is.na(dat$OR_IVW.x),dat$CIL_WM.y,dat$CIL_WM.x)
dat$CIU_WM<-ifelse(is.na(dat$OR_IVW.x),dat$CIU_WM.y,dat$CIU_WM.x)
dat$CI_WM.y<-as.character(dat$CI_WM.y)
dat$CI_WM.x<-as.character(dat$CI_WM.x)
dat$CI_WM<-ifelse(is.na(dat$OR_IVW.x),dat$CI_WM.y,dat$CI_WM.x)
dat$p_WM<-ifelse(is.na(dat$OR_IVW.x),dat$p_WM.y,dat$p_WM.x)

dat$p_adj<-p.adjust(dat$p_IVW, method = "BH", n = length(dat$p_IVW))
write.csv(dat,"cytokine on hospitalized covid combined results updated 2021 jan.csv",row.names =
F)

###any covid
dat8<-read.csv("cytokine on covid mr results using snps at 8 updated 2021 jan.csv",header=T)
dat6<-read.csv("cytokine on covid mr results using snps at 6 updated 2021 jan.csv",header=T)
dat<-merge(dat8,dat6,by='association',all.y=T)
dat$OR_IVW<-ifelse(is.na(dat$OR_IVW.x),dat$OR_IVW.y,dat$OR_IVW.x)
dat$CI_IVW.y<-as.character(dat$CI_IVW.y)
dat$CI_IVW.x<-as.character(dat$CI_IVW.x)
dat$CI_IVW<-ifelse(is.na(dat$OR_IVW.x),dat$CI_IVW.y,dat$CI_IVW.x)
dat$p_IVW<-ifelse(is.na(dat$OR_IVW.x),dat$p_IVW.y,dat$p_IVW.x)
dat$CIL_IVW<-ifelse(is.na(dat$OR_IVW.x),dat$CIL_IVW.y,dat$CIL_IVW.x)
dat$CIU_IVW<-ifelse(is.na(dat$OR_IVW.x),dat$CIU_IVW.y,dat$CIU_IVW.x)
```

```
dat$nsnp<-ifelse(is.na(dat$nsnp.x),dat$nsnp.y,dat$nsnp.x)
dat$p_hetero<-ifelse(is.na(dat$p_hetero.x),dat$p_hetero.y,dat$p_hetero.x)

dat$OR_EGG<-ifelse(is.na(dat$OR_IVW.x),dat$OR_EGG.y,dat$OR_EGG.x)
dat$CIL_EGG<-ifelse(is.na(dat$OR_IVW.x),dat$CIL_EGG.y,dat$CIL_EGG.x)
dat$CIU_EGG<-ifelse(is.na(dat$OR_IVW.x),dat$CIU_EGG.y,dat$CIU_EGG.x)
dat$CI_EGG.y<-as.character(dat$CI_EGG.y)
dat$CI_EGG.x<-as.character(dat$CI_EGG.x)
dat$CI_EGG<-ifelse(is.na(dat$OR_IVW.x),dat$CI_EGG.y,dat$CI_EGG.x)
dat$p_EGG<-ifelse(is.na(dat$OR_IVW.x),dat$p_EGG.y,dat$p_EGG.x)
dat$p_int_EGG<-ifelse(is.na(dat$OR_IVW.x),dat$p_int_EGG.y,dat$p_int_EGG.x)

dat$OR_WM<-ifelse(is.na(dat$OR_IVW.x),dat$OR_WM.y,dat$OR_WM.x)
dat$CIL_WM<-ifelse(is.na(dat$OR_IVW.x),dat$CIL_WM.y,dat$CIL_WM.x)
dat$CIU_WM<-ifelse(is.na(dat$OR_IVW.x),dat$CIU_WM.y,dat$CIU_WM.x)
dat$CI_WM.y<-as.character(dat$CI_WM.y)
dat$CI_WM.x<-as.character(dat$CI_WM.x)
dat$CI_WM<-ifelse(is.na(dat$OR_IVW.x),dat$CI_WM.y,dat$CI_WM.x)
dat$p_WM<-ifelse(is.na(dat$OR_IVW.x),dat$p_WM.y,dat$p_WM.x)

dat$p_adj<-p.adjust(dat$p_IVW, method = "BH", n = length(dat$p_IVW))
write.csv(dat,"cytokine on covid combined results updated 2021 jan.csv",row.names = F)

###combine harmonized datasets
sort<-fread("target_list.csv",header = T )
a<-c("hospitalized covid","covid")
res<-list()
res_final<-data.frame()
for (i in (1:length(sort$exposure)))
{ for (j in (1:length(a)))
{
```

```
  filename<-paste(sort[i,1]," on ",a[j], " vs population harmonized dataset removing palindromic snp.csv",sep="")

  res[[j]]<-read.csv(file=filename, header = TRUE)

  res[[j]]$exposure<-as.character(sort[i,1])

  res[[j]]$outcome<-as.character(a[j])

  res[[j]]<-
res[[j]][c("SNP","effect_allele.exposure","other_allele.exposure","effect_allele.outcome","other_allele.outcome","beta.exposure","se.exposure","pval.exposure","beta.outcome","se.outcome","exposure","outcome")]

}

  ress<-merge(res[[1]],res[[2]],by='SNP',all.y=T)

  #ress<-merge(ress, res[[3]],by='SNP',all.y = T)

  res_final<-rbind(res_final,ress)

}

write.csv(res_final,file="Cytokine on covid summary statistics updated 2021 jan.csv",row.names = F)
```
