# Supplementary material for: Circulating Cytokines and Coronavirus Disease: A Bi-Directional Mendelian Randomization Study
Source: Front Genet. 2021 Jun 7;12:680646. doi: 10.3389/fgene.2021.680646 (PMC8215612; doi:10.3389/fgene.2021.680646)
Supplement: Supplementary file 3 [file Presentation_1.zip › Suppl. Tables.DOCX]

Table S1 Contributing studies in the COVID-19 genome-wide association study.

| Phenotype | Case (total) | Control (total) | Study |  |  |
| --- | --- | --- | --- | --- | --- |
|  |  |  | Name | Case | Control |
| COVID-19 vs. population | 36590 | 1668938 | ACCOuNT_AFR | 57 | 799 |
|  |  |  | BQC19_EUR | 269 | 368 |
|  |  |  | BelCovid_EUR | 485 | 1477 |
|  |  |  | BioVU_EUR | 141 | 70615 |
|  |  |  | CCPM_EUR | 332 | 32375 |
|  |  |  | CU_AFR | 332 | 2610 |
|  |  |  | CU_EUR | 508 | 2149 |
|  |  |  | DECODE_EUR | 4256 | 270934 |
|  |  |  | EstBB_EUR | 1322 | 193774 |
|  |  |  | FinnGen_FIN | 810 | 237901 |
|  |  |  | GCAT_EUR | 253 | 4735 |
|  |  |  | GENCOVID_EUR | 1220 | 2443 |
|  |  |  | GFG_EUR | 147 | 5442 |
|  |  |  | GHS_Freeze_145_EUR | 869 | 112862 |
|  |  |  | GNH_SAS | 1379 | 32785 |
|  |  |  | Genotek_EUR | 676 | 12317 |
|  |  |  | INTERVAL_EUR | 838 | 40994 |
|  |  |  | JapanTaskForce_EAS | 614 | 1705 |
|  |  |  | LGDB_EUR | 275 | 1313 |
|  |  |  | Lifelines_EUR | 244 | 26553 |
|  |  |  | SINAI_COVID_EUR | 330 | 1396 |
|  |  |  | Stanford_EUR | 169 | 190 |
|  |  |  | TOPMed_CHRIS10K_EUR | 92 | 2373 |
|  |  |  | TOPMed_Gardena_EUR | 452 | 458 |
|  |  |  | UCLA_AMR | 169 | 4495 |
|  |  |  | UCLA_EUR | 203 | 17391 |
|  |  |  | UKBB_AFR | 206 | 7691 |
|  |  |  | UKBB_EUR | 6490 | 328577 |
|  |  |  | UKBB_SAS | 309 | 9231 |
|  |  |  | SPGRX_EUR | 362 | 302 |
|  |  |  | PMBB_AFR | 166 | 8436 |
|  |  |  | QGP_ARAB | 700 | 13360 |
|  |  |  | MVP_AFR | 1217 | 6085 |
|  |  |  | MVP_EUR | 1520 | 7600 |
|  |  |  | MVP_HIS | 510 | 2550 |
|  |  |  | Corea_EAS | 108 | 6500 |
|  |  |  | genomicsengland100kgp_EUR | 218 | 62302 |
|  |  |  | Helix_EUR | 178 | 5441 |
|  |  |  | MGI_EUR | 122 | 51458 |
|  |  |  | NTR_EUR | 145 | 5252 |
|  |  |  | PHBB_AFR | 60 | 2445 |
|  |  |  | PHBB_EUR | 151 | 29966 |
|  |  |  | PHBB_HIS | 66 | 2405 |
|  |  |  | Ancestry_EUR | 2417 | 14933 |
|  |  |  | BRACOVID_AMR | 853 | 835 |
|  |  |  | Genetics_COVID19_Korea_EAS | 624 | 6549 |
|  |  |  | idipaz24genetics_EUR | 106 | 75 |
|  |  |  | Amsterdam_UMC_COVID_study_group_EUR | 108 | 1413 |
|  |  |  | HOSTAGE_EUR | 1610 | 2205 |
|  |  |  | SweCovid_EUR | 77 | 3748 |
|  |  |  | genomicc_EAS | 149 | 745 |
|  |  |  | genomicc_EUR | 1676 | 8380 |

(Continued)

| Phenotype | Case (total) | Control (total) | Study |  |  |
| --- | --- | --- | --- | --- | --- |
|  |  |  | Name | Case | Control |
| Hospitalized COVID-19 vs. population | 12888 | 1295966 | BQC19_EUR | 244 | 396 |
|  |  |  | BRACOVID_AMR | 853 | 835 |
|  |  |  | BelCovid_EUR | 363 | 1477 |
|  |  |  | CU_AFR | 304 | 2610 |
|  |  |  | CU_EUR | 453 | 2149 |
|  |  |  | EstBB_EUR | 90 | 196339 |
|  |  |  | FinnGen_FIN | 106 | 238605 |
|  |  |  | GENCOVID_EUR | 893 | 2443 |
|  |  |  | GHS_Freeze_145_EUR | 180 | 112862 |
|  |  |  | GNH_SAS | 115 | 34049 |
|  |  |  | Genetics_COVID19_Korea_EAS | 624 | 6549 |
|  |  |  | JapanTaskForce_EAS | 572 | 1705 |
|  |  |  | LGDB_EUR | 57 | 1531 |
|  |  |  | UCLA_AMR | 95 | 4569 |
|  |  |  | UCLA_EUR | 80 | 17514 |
|  |  |  | UKBB_AFR | 71 | 7691 |
|  |  |  | UKBB_EUR | 1670 | 328577 |
|  |  |  | UKBB_SAS | 71 | 9231 |
|  |  |  | idipaz24genetics_EUR | 106 | 75 |
|  |  |  | Amsterdam_UMC_COVID_study_group_EUR | 108 | 1413 |
|  |  |  | SPGRX_EUR | 311 | 302 |
|  |  |  | DECODE_EUR | 89 | 274322 |
|  |  |  | PMBB_AFR | 66 | 8536 |
|  |  |  | QGP_ARAB | 60 | 13360 |
|  |  |  | MVP_AFR | 349 | 1745 |
|  |  |  | MVP_EUR | 436 | 2180 |
|  |  |  | MVP_HIS | 117 | 585 |
|  |  |  | Corea_EAS | 69 | 6500 |
|  |  |  | HOSTAGE_EUR | 1610 | 2205 |
|  |  |  | BoSCO_EUR | 212 | 512 |
|  |  |  | FHoGID_EUR | 362 | 259 |
|  |  |  | Ancestry_EUR | 250 | 1967 |
|  |  |  | SweCovid_EUR | 77 | 3748 |
|  |  |  | genomicc_EAS | 149 | 745 |
|  |  |  | genomicc_EUR | 1676 | 8380 |

Table S2 F statistics of genetic predictors of 41 cytokines.

| Exposure | F-statistics at 5× 10^-8^ | F-statistics at 5× 10^-6^ |
| --- | --- | --- |
| bNGF | - | 24.0 |
| CTACK | 67.8 | - |
| Eotaxin | 86.7 | - |
| FGFBasic | - | 22.9 |
| GCSF | - | 23.2 |
| GROa | 110.1 | - |
| HGF | 49.0 | - |
| IFNg | - | 23.6 |
| IL-10 | 125.5 | - |
| IL12p70 | 88.8 | - |
| IL-13 | 120.2 | - |
| IL-16 | 67.9 | - |
| IL-17 | - | 23.9 |
| IL-18 | 59.9 | - |
| IL1b | - | 18.6 |
| IL1ra | - | 22.2 |
| IL-2 | - | 22.7 |
| IL2ra | 135.4 | - |
| IL-4 | - | 23.4 |
| IL-5 | - | 26.0 |
| IL-6 | - | 24.0 |
| IL-7 | 169.8 | 34.4 |
| IL-8 | - | 24.0 |
| IL-9 | - | 22.3 |
| IP10 | 31.6 | - |
| MCP1 | 81.2 | - |
| MCP3 | - | 23.8 |
| MCSF | - | 23.4 |
| MIF | - | 25.2 |
| MIG | - | 24.9 |
| MIP1a | - | 22.1 |
| MIP1b | 82.3 | - |
| PDGFbb | 69.0 | - |
| RANTES | - | 24.3 |
| SCF | 40.2 | - |
| SCGFb | 60.5 | - |
| SDF1a | - | 18.7 |
| TNFa | - | 23.5 |
| TNFb | 56.8 | - |
| TRAIL | 122.4 | - |
| VEGF | 88.5 | - |

Table S3 F statistics of genetic predictors of three phenotypes of COVID-19infections.

| Exposure | F statistics |
| --- | --- |
| COVID-19 vs. population | 60.5 |
| Hospitalized COVID-19 vs. population | 64.8 |

Table S4 Effects of 41 circulating cytokines on any COVID-19 using Mendelian Randomization with different methods (leave out 23 and me).

| Exposure | No. SNP | IVW |  |  |  | MR-Egger |  |  |  | WM |  |  |
| --- | --- | --- | --- | --- | --- | --- | --- | --- | --- | --- | --- | --- |
|  |  | OR | 95% CI | P value | P_corrected | OR | 95% CI | P value | P_intercept | OR | 95% CI | P value |
| bNGF | 7 | 0.99 | 0.94, 1.05 | 0.83 | 0.94 | 1.22 | 0.92, 1.61 | 0.17 | 0.15 | 0.99 | 0.92, 1.06 | 0.76 |
| CTACK | 4 | 1.03 | 0.99, 1.08 | 0.16 | 0.94 | 1.00 | 0.90, 1.12 | 0.94 | 0.60 | 1.02 | 0.97, 1.08 | 0.39 |
| Eotaxin | 6 | 0.99 | 0.91, 1.07 | 0.72 | 0.94 | 1.05 | 0.85, 1.30 | 0.65 | 0.51 | 1.00 | 0.94, 1.07 | 0.90 |
| FGFBasic | 6 | 1.05 | 0.97, 1.14 | 0.21 | 0.94 | 1.06 | 0.89, 1.26 | 0.54 | 0.98 | 1.06 | 0.95, 1.18 | 0.31 |
| GCSF | 9 | 0.99 | 0.93, 1.06 | 0.79 | 0.94 | 1.01 | 0.92, 1.11 | 0.79 | 0.52 | 1.03 | 0.96, 1.10 | 0.43 |
| GROa | 5 | 1.00 | 0.97, 1.03 | 0.87 | 0.95 | 1.02 | 0.93, 1.11 | 0.71 | 0.74 | 1.01 | 0.97, 1.04 | 0.75 |
| HGF | 2 | 0.92 | 0.84, 1.01 | 0.09 | 0.94 | - | - | - | - | - | - | - |
| IFNg | 12 | 1.02 | 0.95, 1.09 | 0.62 | 0.94 | 0.92 | 0.82, 1.03 | 0.15 | 0.04 | 1.04 | 0.96, 1.12 | 0.38 |
| IL10 | 3 | 1.02 | 0.97, 1.08 | 0.39 | 0.94 | 1.06 | 0.93, 1.20 | 0.39 | 0.59 | 1.03 | 0.97, 1.09 | 0.32 |
| IL12p70 | 11 | 1.01 | 0.98, 1.05 | 0.56 | 0.94 | 1.03 | 0.96, 1.11 | 0.40 | 0.53 | 1.02 | 0.98, 1.06 | 0.36 |
| IL13 | 2 | 1.01 | 0.97, 1.05 | 0.75 | 0.94 | - | - | - | - | - | - | - |
| IL16 | 3 | 0.97 | 0.94, 1.01 | 0.12 | 0.94 | 0.96 | 0.91, 1.02 | 0.18 | 0.67 | 0.97 | 0.93, 1.01 | 0.10 |
| IL17 | 9 | 1.01 | 0.95, 1.08 | 0.75 | 0.94 | 1.08 | 0.95, 1.24 | 0.24 | 0.24 | 0.99 | 0.91, 1.08 | 0.90 |
| IL18 | 3 | 0.98 | 0.93, 1.03 | 0.36 | 0.94 | 1.30 | 0.30, 5.67 | 0.73 | 0.71 | 0.94 | 0.88, 1.02 | 0.12 |
| IL1b | 5 | 1.05 | 0.96, 1.15 | 0.28 | 0.94 | 1.10 | 0.93, 1.32 | 0.27 | 0.51 | 1.05 | 0.94, 1.18 | 0.34 |
| IL1ra | 6 | 1.00 | 0.94, 1.07 | 0.95 | 0.95 | 1.11 | 0.96, 1.29 | 0.17 | 0.14 | 1.02 | 0.95, 1.11 | 0.56 |
| IL2 | 10 | 1.02 | 0.97, 1.06 | 0.49 | 0.94 | 1.00 | 0.91, 1.09 | 0.93 | 0.62 | 1.00 | 0.95, 1.06 | 0.90 |
| IL2ra | 2 | 0.98 | 0.94, 1.02 | 0.37 | 0.94 | - | - | - | - | - | - | - |
| IL4 | 10 | 0.97 | 0.92, 1.03 | 0.35 | 0.94 | 0.94 | 0.85, 1.04 | 0.20 | 0.37 | 0.96 | 0.88, 1.03 | 0.25 |
| IL5 | 5 | 0.99 | 0.93, 1.05 | 0.74 | 0.94 | 0.93 | 0.80, 1.10 | 0.41 | 0.44 | 0.98 | 0.91, 1.06 | 0.60 |
| IL6 | 6 | 0.94 | 0.87, 1.02 | 0.14 | 0.94 | 1.00 | 0.87, 1.15 | 1.00 | 0.34 | 0.95 | 0.86, 1.04 | 0.28 |
| IL7 | 12 | 1.00 | 0.97, 1.03 | 0.94 | 0.95 | 1.04 | 0.97, 1.12 | 0.29 | 0.25 | 1.01 | 0.97, 1.06 | 0.61 |
| IL8 | 4 | 0.98 | 0.93, 1.04 | 0.57 | 0.94 | 0.98 | 0.89, 1.08 | 0.71 | 0.97 | 0.99 | 0.92, 1.06 | 0.74 |
| IL9 | 5 | 1.03 | 0.94, 1.14 | 0.51 | 0.94 | 1.11 | 0.89, 1.38 | 0.36 | 0.48 | 0.99 | 0.91, 1.08 | 0.87 |
| IP10 | 10 | 1.01 | 0.96, 1.07 | 0.63 | 0.94 | 1.06 | 0.95, 1.18 | 0.28 | 0.33 | 1.02 | 0.96, 1.08 | 0.45 |
| MCP1 | 6 | 1.05 | 0.99, 1.12 | 0.11 | 0.94 | 1.04 | 0.87, 1.24 | 0.67 | 0.88 | 1.01 | 0.95, 1.08 | 0.72 |
| MCP3 | 3 | 0.97 | 0.92, 1.03 | 0.30 | 0.94 | 0.98 | 0.74, 1.29 | 0.89 | 0.94 | 0.99 | 0.93, 1.07 | 0.88 |
| MCSF | 7 | 1.01 | 0.96, 1.07 | 0.68 | 0.94 | 1.06 | 0.96, 1.17 | 0.27 | 0.30 | 1.03 | 0.97, 1.09 | 0.34 |
| MIF | 6 | 0.99 | 0.94, 1.03 | 0.54 | 0.94 | 0.98 | 0.92, 1.05 | 0.61 | 0.89 | 0.99 | 0.94, 1.05 | 0.85 |
| MIG | 12 | 1.02 | 0.98, 1.06 | 0.35 | 0.94 | 0.97 | 0.90, 1.04 | 0.38 | 0.12 | 1.04 | 0.99, 1.10 | 0.15 |
| MIP1a | 6 | 0.97 | 0.91, 1.03 | 0.29 | 0.94 | 0.93 | 0.77, 1.12 | 0.42 | 0.64 | 0.96 | 0.89, 1.03 | 0.25 |
| MIP1b | 45 | 0.97 | 0.96, 0.99 | 0.01 | 0.35 | 0.96 | 0.93, 1.00 | 0.05 | 0.52 | 0.96 | 0.94, 0.99 | 0.01 |
| PDGFbb | 7 | 1.02 | 0.97, 1.07 | 0.44 | 0.94 | 1.01 | 0.91, 1.12 | 0.82 | 0.88 | 1.01 | 0.96, 1.07 | 0.65 |
| RANTES | 9 | 1.00 | 0.95, 1.05 | 0.93 | 0.95 | 0.96 | 0.83, 1.11 | 0.55 | 0.54 | 1.00 | 0.94, 1.07 | 0.91 |
| SCF | 2 | 1.04 | 0.93, 1.17 | 0.48 | 0.94 | - | - | - | - | - | - | - |
| SCGFb | 5 | 1.00 | 0.95, 1.05 | 0.88 | 0.95 | 1.00 | 0.89, 1.12 | 0.98 | 0.97 | 0.97 | 0.92, 1.03 | 0.37 |
| SDF1a | 9 | 0.98 | 0.91, 1.04 | 0.47 | 0.94 | 1.00 | 0.90, 1.11 | 0.99 | 0.57 | 0.98 | 0.89, 1.07 | 0.59 |
| TNFa | 4 | 1.01 | 0.93, 1.10 | 0.75 | 0.94 | 1.01 | 0.83, 1.24 | 0.88 | 0.99 | 1.01 | 0.93, 1.09 | 0.86 |
| TNFb | 3 | 1.00 | 0.97, 1.03 | 0.82 | 0.94 | 1.05 | 0.91, 1.23 | 0.49 | 0.51 | 1.01 | 0.97, 1.04 | 0.71 |
| TRAIL | 18 | 1.02 | 0.99, 1.05 | 0.24 | 0.94 | 1.04 | 1.00, 1.09 | 0.04 | 0.11 | 1.02 | 0.98, 1.05 | 0.29 |
| VEGF | 16 | 1.01 | 0.98, 1.03 | 0.66 | 0.94 | 1.03 | 0.98, 1.08 | 0.31 | 0.35 | 1.02 | 0.98, 1.05 | 0.36 |

Table S5 Effects of 41 circulating cytokines on hospitalized COVID-19 using Mendelian Randomization with different methods (leave out 23 and me).

| Exposure | No. SNP | IVW |  |  |  | MR-Egger |  |  |  | WM |  |  |
| --- | --- | --- | --- | --- | --- | --- | --- | --- | --- | --- | --- | --- |
|  |  | OR | 95% CI | P value | P_corrected | OR | 95% CI | P value | P_intercept | OR | 95% CI | P value |
| bNGF | 7 | 1.01 | 0.89, 1.14 | 0.93 | 0.97 | 1.26 | 0.66, 2.40 | 0.47 | 0.48 | 0.99 | 0.86, 1.14 | 0.88 |
| CTACK | 4 | 0.98 | 0.86, 1.11 | 0.73 | 0.93 | 0.83 | 0.63, 1.08 | 0.17 | 0.18 | 1.01 | 0.91, 1.12 | 0.82 |
| Eotaxin | 5 | 0.95 | 0.85, 1.06 | 0.35 | 0.88 | 0.92 | 0.68, 1.23 | 0.56 | 0.79 | 0.98 | 0.87, 1.10 | 0.75 |
| FGFBasic | 5 | 0.90 | 0.75, 1.09 | 0.28 | 0.88 | 1.64 | 0.61, 4.39 | 0.32 | 0.22 | 0.93 | 0.74, 1.17 | 0.55 |
| GCSF | 8 | 1.01 | 0.90, 1.14 | 0.85 | 0.97 | 1.08 | 0.89, 1.31 | 0.45 | 0.42 | 1.08 | 0.92, 1.27 | 0.35 |
| GROa | 4 | 0.98 | 0.93, 1.04 | 0.60 | 0.93 | 1.05 | 0.88, 1.25 | 0.62 | 0.47 | 0.99 | 0.92, 1.06 | 0.72 |
| HGF | 2 | 0.92 | 0.77, 1.09 | 0.33 | 0.88 | - | - | - | - | - | - | - |
| IFNg | 11 | 1.01 | 0.90, 1.13 | 0.88 | 0.97 | 1.00 | 0.80, 1.26 | 0.97 | 0.97 | 1.00 | 0.86, 1.16 | 0.95 |
| IL10 | 3 | 1.05 | 0.95, 1.17 | 0.35 | 0.88 | 0.94 | 0.73, 1.20 | 0.61 | 0.31 | 1.03 | 0.92, 1.16 | 0.56 |
| IL12p70 | 10 | 1.02 | 0.95, 1.11 | 0.54 | 0.93 | 1.02 | 0.87, 1.20 | 0.79 | 0.97 | 1.02 | 0.94, 1.10 | 0.70 |
| IL13 | 2 | 1.00 | 0.92, 1.09 | 0.96 | 0.97 | - | - | - | - | - | - | - |
| IL16 | 3 | 0.95 | 0.89, 1.01 | 0.10 | 0.88 | 0.96 | 0.87, 1.06 | 0.43 | 0.73 | 0.95 | 0.89, 1.02 | 0.14 |
| IL17 | 8 | 0.99 | 0.86, 1.15 | 0.95 | 0.97 | 1.15 | 0.81, 1.63 | 0.43 | 0.36 | 0.97 | 0.82, 1.16 | 0.75 |
| IL18 | 3 | 0.95 | 0.86, 1.05 | 0.33 | 0.88 | 1.90 | 0.06, 60.03 | 0.72 | 0.69 | 0.88 | 0.75, 1.02 | 0.08 |
| IL1b | 5 | 1.06 | 0.90, 1.24 | 0.49 | 0.93 | 1.13 | 0.82, 1.54 | 0.46 | 0.65 | 1.06 | 0.87, 1.29 | 0.55 |
| IL1ra | 6 | 1.05 | 0.93, 1.18 | 0.43 | 0.88 | 1.10 | 0.79, 1.53 | 0.56 | 0.75 | 1.07 | 0.93, 1.24 | 0.33 |
| IL2 | 9 | 1.01 | 0.93, 1.10 | 0.75 | 0.93 | 0.98 | 0.83, 1.16 | 0.83 | 0.67 | 0.99 | 0.89, 1.10 | 0.91 |
| IL2ra | 2 | 0.95 | 0.88, 1.02 | 0.18 | 0.88 | - | - | - | - | - | - | - |
| IL4 | 9 | 1.06 | 0.95, 1.19 | 0.30 | 0.88 | 1.01 | 0.83, 1.24 | 0.91 | 0.58 | 1.00 | 0.86, 1.16 | 0.99 |
| IL5 | 4 | 0.98 | 0.86, 1.12 | 0.78 | 0.93 | 0.92 | 0.64, 1.31 | 0.63 | 0.68 | 1.02 | 0.87, 1.19 | 0.84 |
| IL6 | 5 | 0.91 | 0.78, 1.06 | 0.22 | 0.88 | 1.00 | 0.73, 1.37 | 1.00 | 0.49 | 0.91 | 0.75, 1.11 | 0.35 |
| IL7 | 10 | 1.00 | 0.93, 1.07 | 0.97 | 0.97 | 0.93 | 0.79, 1.08 | 0.34 | 0.29 | 0.98 | 0.89, 1.07 | 0.61 |
| IL8 | 3 | 1.02 | 0.91, 1.14 | 0.78 | 0.93 | 1.00 | 0.84, 1.21 | 0.96 | 0.88 | 1.01 | 0.89, 1.15 | 0.88 |
| IL9 | 5 | 1.03 | 0.89, 1.19 | 0.69 | 0.93 | 1.19 | 0.89, 1.60 | 0.24 | 0.26 | 0.99 | 0.84, 1.17 | 0.93 |
| IP10 | 10 | 1.02 | 0.93, 1.12 | 0.67 | 0.93 | 1.09 | 0.92, 1.29 | 0.32 | 0.36 | 0.99 | 0.88, 1.12 | 0.89 |
| MCP1 | 6 | 1.10 | 0.88, 1.39 | 0.39 | 0.88 | 0.91 | 0.49, 1.70 | 0.78 | 0.52 | 1.04 | 0.92, 1.18 | 0.54 |
| MCP3 | 3 | 0.96 | 0.85, 1.07 | 0.43 | 0.88 | 0.92 | 0.53, 1.58 | 0.76 | 0.88 | 0.98 | 0.85, 1.12 | 0.77 |
| MCSF | 5 | 1.04 | 0.95, 1.13 | 0.42 | 0.88 | 1.09 | 0.93, 1.28 | 0.29 | 0.45 | 1.01 | 0.90, 1.13 | 0.90 |
| MIF | 5 | 0.96 | 0.88, 1.04 | 0.32 | 0.88 | 0.98 | 0.87, 1.10 | 0.70 | 0.67 | 0.97 | 0.88, 1.07 | 0.53 |
| MIG | 11 | 0.97 | 0.90, 1.04 | 0.36 | 0.88 | 0.83 | 0.72, 0.96 | 0.01 | 0.02 | 0.94 | 0.84, 1.04 | 0.24 |
| MIP1a | 6 | 0.94 | 0.83, 1.06 | 0.32 | 0.88 | 0.90 | 0.62, 1.31 | 0.59 | 0.83 | 0.97 | 0.84, 1.13 | 0.71 |
| MIP1b | 38 | 0.93 | 0.89, 0.98 | 0.01 | 0.24 | 0.90 | 0.82, 0.99 | 0.03 | 0.44 | 0.92 | 0.87, 0.98 | 0.01 |
| PDGFbb | 6 | 0.98 | 0.83, 1.15 | 0.77 | 0.93 | 0.99 | 0.68, 1.44 | 0.96 | 0.93 | 1.01 | 0.91, 1.12 | 0.86 |
| RANTES | 9 | 1.02 | 0.90, 1.15 | 0.80 | 0.93 | 0.88 | 0.62, 1.26 | 0.50 | 0.41 | 1.02 | 0.89, 1.16 | 0.80 |
| SCF | 2 | 1.21 | 0.97, 1.51 | 0.10 | 0.88 | - | - | - | - | - | - | - |
| SCGFb | 4 | 1.04 | 0.88, 1.23 | 0.65 | 0.93 | 0.82 | 0.59, 1.13 | 0.23 | 0.11 | 1.02 | 0.90, 1.16 | 0.77 |
| SDF1a | 7 | 0.89 | 0.77, 1.02 | 0.10 | 0.88 | 0.78 | 0.63, 0.98 | 0.03 | 0.16 | 0.85 | 0.71, 1.02 | 0.09 |
| TNFa | 4 | 0.97 | 0.87, 1.10 | 0.66 | 0.93 | 0.96 | 0.74, 1.25 | 0.77 | 0.91 | 0.94 | 0.81, 1.09 | 0.44 |
| TNFb | 3 | 1.03 | 0.97, 1.09 | 0.30 | 0.88 | 0.88 | 0.70, 1.11 | 0.29 | 0.18 | 1.03 | 0.97, 1.10 | 0.34 |
| TRAIL | 14 | 1.01 | 0.96, 1.06 | 0.75 | 0.93 | 1.03 | 0.96, 1.11 | 0.36 | 0.32 | 1.04 | 0.97, 1.11 | 0.26 |
| VEGF | 15 | 0.99 | 0.94, 1.04 | 0.72 | 0.93 | 1.01 | 0.91, 1.12 | 0.88 | 0.70 | 0.99 | 0.92, 1.06 | 0.77 |

Table S6 Effects of 41 circulating cytokines on any COVID-19 using Mendelian Randomization with different methods (leave out ukb).

| Exposure | No. SNP | IVW | | | | MR-Egger | | | | Wm | | |
| --- | --- | --- | --- | --- | --- | --- | --- | --- | --- | --- | --- | --- |
|  |  | OR | 95% CI | P value | P_corrected | OR | 95% CI | P value | P_intercept | OR | 95% CI | P value |
| CTACK | 4 | 1.02 | 0.97, 1.07 | 0.47 | 0.85 | 0.98 | 0.87, 1.10 | 0.69 | 0.44 | 1.00 | 0.95, 1.06 | 0.90 |
| Eotaxin | 6 | 1.01 | 0.95, 1.09 | 0.71 | 0.93 | 1.09 | 0.91, 1.30 | 0.35 | 0.39 | 1.01 | 0.94, 1.09 | 0.81 |
| FGFBasic | 5 | 1.08 | 0.96, 1.21 | 0.21 | 0.85 | 1.18 | 0.65, 2.14 | 0.58 | 0.76 | 1.05 | 0.90, 1.22 | 0.52 |
| GCSF | 9 | 1.00 | 0.94, 1.06 | 0.99 | 0.99 | 1.01 | 0.92, 1.11 | 0.86 | 0.79 | 1.00 | 0.92, 1.08 | 0.99 |
| GROa | 4 | 1.02 | 0.97, 1.06 | 0.46 | 0.85 | 1.04 | 0.90, 1.20 | 0.61 | 0.76 | 1.03 | 0.98, 1.07 | 0.24 |
| HGF | 2 | 0.93 | 0.84, 1.04 | 0.22 | 0.85 | - | - | - | - | - | - | - |
| IFNg | 11 | 1.05 | 0.96, 1.14 | 0.28 | 0.85 | 0.93 | 0.80, 1.08 | 0.33 | 0.07 | 1.04 | 0.95, 1.15 | 0.36 |
| IL10 | 3 | 1.06 | 0.99, 1.13 | 0.07 | 0.85 | 1.12 | 0.97, 1.30 | 0.12 | 0.40 | 1.07 | 1.00, 1.15 | 0.04 |
| IL12p70 | 10 | 1.03 | 0.99, 1.07 | 0.20 | 0.85 | 1.08 | 0.99, 1.17 | 0.08 | 0.20 | 1.04 | 0.99, 1.09 | 0.12 |
| IL13 | 2 | 1.02 | 0.97, 1.07 | 0.40 | 0.85 | - | - | - | - | - | - | - |
| IL16 | 3 | 0.97 | 0.93, 1.01 | 0.17 | 0.85 | 0.95 | 0.89, 1.01 | 0.12 | 0.38 | 0.96 | 0.92, 1.01 | 0.11 |
| IL17 | 8 | 0.99 | 0.90, 1.09 | 0.88 | 0.95 | 1.10 | 0.87, 1.41 | 0.42 | 0.35 | 1.01 | 0.90, 1.13 | 0.87 |
| IL18 | 2 | 0.99 | 0.93, 1.06 | 0.84 | 0.93 | - | - | - | - | - | - | - |
| IL1b | 5 | 1.02 | 0.92, 1.13 | 0.66 | 0.93 | 1.10 | 0.91, 1.35 | 0.33 | 0.37 | 1.02 | 0.90, 1.15 | 0.80 |
| IL1ra | 6 | 1.00 | 0.93, 1.07 | 0.98 | 0.99 | 1.06 | 0.88, 1.27 | 0.56 | 0.53 | 1.02 | 0.93, 1.11 | 0.75 |
| IL2 | 9 | 1.02 | 0.96, 1.07 | 0.52 | 0.86 | 0.98 | 0.88, 1.09 | 0.75 | 0.46 | 1.02 | 0.96, 1.09 | 0.53 |
| IL2ra | 2 | 1.01 | 0.97, 1.06 | 0.59 | 0.93 | - | - | - | - | - | - | - |
| IL4 | 10 | 0.97 | 0.90, 1.03 | 0.32 | 0.85 | 0.92 | 0.81, 1.04 | 0.17 | 0.32 | 0.94 | 0.86, 1.03 | 0.19 |
| IL5 | 4 | 1.04 | 0.96, 1.13 | 0.32 | 0.85 | 1.00 | 0.73, 1.36 | 0.98 | 0.77 | 1.01 | 0.91, 1.12 | 0.86 |
| IL6 | 6 | 0.96 | 0.85, 1.08 | 0.49 | 0.85 | 1.05 | 0.83, 1.31 | 0.70 | 0.38 | 0.99 | 0.88, 1.12 | 0.88 |
| IL7 | 11 | 1.01 | 0.96, 1.06 | 0.75 | 0.93 | 1.09 | 0.99, 1.20 | 0.06 | 0.06 | 1.05 | 0.99, 1.11 | 0.10 |
| IL8 | 4 | 0.97 | 0.91, 1.04 | 0.40 | 0.85 | 0.93 | 0.83, 1.05 | 0.23 | 0.38 | 0.96 | 0.89, 1.04 | 0.33 |
| IL9 | 5 | 1.08 | 0.96, 1.21 | 0.22 | 0.85 | 1.20 | 0.93, 1.55 | 0.17 | 0.35 | 1.02 | 0.92, 1.13 | 0.67 |
| IP10 | 10 | 1.02 | 0.96, 1.08 | 0.48 | 0.85 | 1.06 | 0.95, 1.18 | 0.29 | 0.42 | 1.03 | 0.96, 1.10 | 0.46 |
| MCP1 | 6 | 1.09 | 1.01, 1.17 | 0.03 | 0.85 | 1.02 | 0.84, 1.26 | 0.82 | 0.53 | 1.08 | 1.00, 1.17 | 0.06 |
| MCP3 | 3 | 0.99 | 0.92, 1.05 | 0.66 | 0.93 | 1.02 | 0.82, 1.26 | 0.87 | 0.76 | 1.00 | 0.92, 1.08 | 0.92 |
| MCSF | 6 | 1.02 | 0.97, 1.08 | 0.39 | 0.85 | 1.05 | 0.96, 1.15 | 0.31 | 0.52 | 1.01 | 0.95, 1.08 | 0.68 |
| MIF | 5 | 0.99 | 0.94, 1.05 | 0.84 | 0.93 | 1.01 | 0.94, 1.09 | 0.76 | 0.54 | 1.01 | 0.95, 1.08 | 0.71 |
| MIG | 10 | 0.99 | 0.95, 1.04 | 0.84 | 0.93 | 0.96 | 0.87, 1.06 | 0.39 | 0.38 | 1.01 | 0.94, 1.07 | 0.87 |
| MIP1a | 6 | 0.99 | 0.92, 1.06 | 0.71 | 0.93 | 0.93 | 0.75, 1.16 | 0.54 | 0.60 | 0.99 | 0.90, 1.08 | 0.80 |
| MIP1b | 38 | 0.98 | 0.95, 1.00 | 0.10 | 0.85 | 0.96 | 0.91, 1.01 | 0.09 | 0.34 | 0.98 | 0.94, 1.01 | 0.19 |
| PDGFbb | 6 | 1.01 | 0.96, 1.07 | 0.73 | 0.93 | 1.03 | 0.92, 1.16 | 0.61 | 0.69 | 1.01 | 0.95, 1.08 | 0.69 |
| RANTES | 9 | 1.02 | 0.95, 1.09 | 0.65 | 0.93 | 1.01 | 0.82, 1.23 | 0.95 | 0.92 | 1.03 | 0.95, 1.11 | 0.49 |
| SCF | 2 | 1.06 | 0.93, 1.22 | 0.38 | 0.85 | - | - | - | - | - | - | - |
| SCGFb | 4 | 0.96 | 0.90, 1.02 | 0.18 | 0.85 | 0.87 | 0.75, 1.01 | 0.06 | 0.15 | 0.95 | 0.89, 1.03 | 0.22 |
| SDF1a | 8 | 0.97 | 0.89, 1.06 | 0.50 | 0.85 | 1.03 | 0.89, 1.19 | 0.70 | 0.33 | 0.99 | 0.88, 1.10 | 0.81 |
| TNFa | 4 | 1.01 | 0.92, 1.11 | 0.77 | 0.93 | 0.99 | 0.80, 1.23 | 0.91 | 0.78 | 1.00 | 0.91, 1.09 | 0.96 |
| TNFb | 3 | 1.00 | 0.97, 1.04 | 0.95 | 0.99 | 1.02 | 0.88, 1.18 | 0.79 | 0.80 | 1.00 | 0.96, 1.04 | 0.94 |
| TRAIL | 16 | 1.01 | 0.98, 1.05 | 0.46 | 0.85 | 1.05 | 1.00, 1.10 | 0.06 | 0.06 | 1.03 | 0.99, 1.07 | 0.21 |
| VEGF | 15 | 1.02 | 0.99, 1.06 | 0.13 | 0.85 | 1.05 | 0.99, 1.11 | 0.13 | 0.39 | 1.04 | 1.00, 1.08 | 0.09 |

Table S7 Effects of 41 circulating cytokines on hospitalized COVID-19 using Mendelian Randomization with different methods (leave out ukb).

| Outcome | No. SNP | IVW | | | | MR-Egger | | | | WM | | |
| --- | --- | --- | --- | --- | --- | --- | --- | --- | --- | --- | --- | --- |
|  |  | Beta | 95% CI | P value | P_corrected | Beta | 95% CI | P value | P_intercept | Beta | 95% CI | P value |
| bNGF | 6 | 0.96 | 0.84, 1.10 | 0.56 | 0.98 | 1.17 | 0.61, 2.24 | 0.63 | 0.54 | 0.93 | 0.78, 1.10 | 0.40 |
| CTACK | 4 | 0.99 | 0.90, 1.09 | 0.86 | 0.98 | 0.87 | 0.69, 1.11 | 0.26 | 0.25 | 1.01 | 0.90, 1.14 | 0.84 |
| Eotaxin | 5 | 1.02 | 0.90, 1.15 | 0.76 | 0.98 | 0.93 | 0.69, 1.26 | 0.64 | 0.52 | 1.03 | 0.90, 1.18 | 0.66 |
| FGFBasic | 5 | 0.89 | 0.70, 1.12 | 0.33 | 0.94 | 2.24 | 0.72, 6.93 | 0.16 | 0.10 | 0.97 | 0.74, 1.28 | 0.84 |
| GCSF | 7 | 0.99 | 0.84, 1.18 | 0.95 | 0.98 | 1.05 | 0.73, 1.53 | 0.78 | 0.73 | 1.09 | 0.87, 1.37 | 0.45 |
| GROa | 4 | 1.01 | 0.95, 1.09 | 0.69 | 0.98 | 1.08 | 0.88, 1.32 | 0.46 | 0.53 | 1.02 | 0.94, 1.10 | 0.64 |
| HGF | 2 | 0.90 | 0.74, 1.10 | 0.32 | 0.94 | - | - | - | - | - | - | - |
| IFNg | 10 | 1.07 | 0.94, 1.22 | 0.31 | 0.94 | 1.17 | 0.90, 1.51 | 0.25 | 0.46 | 1.08 | 0.91, 1.28 | 0.38 |
| IL10 | 3 | 1.11 | 0.98, 1.26 | 0.09 | 0.90 | 1.10 | 0.82, 1.46 | 0.53 | 0.91 | 1.10 | 0.97, 1.26 | 0.14 |
| IL12p70 | 10 | 1.06 | 0.98, 1.15 | 0.14 | 0.90 | 1.11 | 0.94, 1.31 | 0.21 | 0.54 | 1.08 | 0.98, 1.19 | 0.11 |
| IL13 | 2 | 1.05 | 0.94, 1.17 | 0.39 | 0.94 | - | - | - | - | - | - | - |
| IL16 | 3 | 0.96 | 0.89, 1.03 | 0.23 | 0.93 | 0.98 | 0.87, 1.09 | 0.70 | 0.62 | 0.96 | 0.89, 1.04 | 0.30 |
| IL17 | 7 | 0.95 | 0.77, 1.16 | 0.60 | 0.98 | 1.50 | 0.87, 2.56 | 0.14 | 0.08 | 1.00 | 0.80, 1.26 | 0.97 |
| IL18 | 2 | 0.95 | 0.83, 1.09 | 0.44 | 0.98 | - | - | - | - | - | - | - |
| IL1b | 5 | 0.95 | 0.80, 1.14 | 0.61 | 0.98 | 1.07 | 0.76, 1.51 | 0.71 | 0.45 | 0.99 | 0.79, 1.25 | 0.96 |
| IL1ra | 6 | 1.06 | 0.93, 1.21 | 0.39 | 0.94 | 0.90 | 0.65, 1.25 | 0.54 | 0.29 | 1.02 | 0.86, 1.21 | 0.79 |
| IL2 | 9 | 1.00 | 0.89, 1.12 | 0.93 | 0.98 | 0.96 | 0.74, 1.24 | 0.77 | 0.77 | 1.01 | 0.89, 1.14 | 0.88 |
| IL2ra | 2 | 0.99 | 0.91, 1.08 | 0.84 | 0.98 | - | - | - | - | - | - | - |
| IL4 | 7 | 1.05 | 0.88, 1.24 | 0.60 | 0.98 | 1.08 | 0.59, 1.97 | 0.81 | 0.93 | 1.05 | 0.85, 1.30 | 0.66 |
| IL5 | 3 | 1.05 | 0.89, 1.23 | 0.56 | 0.98 | 0.94 | 0.52, 1.72 | 0.85 | 0.72 | 1.02 | 0.84, 1.23 | 0.84 |
| IL6 | 5 | 0.90 | 0.75, 1.07 | 0.24 | 0.93 | 1.02 | 0.71, 1.48 | 0.90 | 0.42 | 0.93 | 0.75, 1.17 | 0.54 |
| IL7 | 9 | 1.01 | 0.92, 1.12 | 0.80 | 0.98 | 0.96 | 0.76, 1.21 | 0.70 | 0.58 | 1.06 | 0.94, 1.18 | 0.36 |
| IL8 | 3 | 1.01 | 0.88, 1.15 | 0.90 | 0.98 | 0.96 | 0.77, 1.19 | 0.68 | 0.53 | 1.00 | 0.86, 1.16 | 0.97 |
| IL9 | 4 | 0.97 | 0.82, 1.15 | 0.72 | 0.98 | 0.94 | 0.63, 1.39 | 0.74 | 0.84 | 1.03 | 0.83, 1.28 | 0.76 |
| IP10 | 7 | 1.00 | 0.88, 1.14 | 0.98 | 0.98 | 1.06 | 0.78, 1.44 | 0.70 | 0.67 | 0.95 | 0.80, 1.11 | 0.50 |
| MCP1 | 6 | 1.15 | 0.90, 1.47 | 0.25 | 0.93 | 0.87 | 0.46, 1.64 | 0.67 | 0.34 | 1.07 | 0.93, 1.22 | 0.34 |
| MCP3 | 2 | 1.00 | 0.85, 1.17 | 0.96 | 0.98 | - | - | - | - | - | - | - |
| MCSF | 3 | 1.10 | 0.97, 1.26 | 0.14 | 0.90 | 1.13 | 0.85, 1.50 | 0.39 | 0.84 | 1.08 | 0.92, 1.27 | 0.35 |
| MIF | 5 | 0.94 | 0.85, 1.04 | 0.25 | 0.93 | 1.01 | 0.89, 1.15 | 0.89 | 0.14 | 0.98 | 0.88, 1.09 | 0.74 |
| MIG | 7 | 1.01 | 0.89, 1.14 | 0.91 | 0.98 | 0.75 | 0.48, 1.16 | 0.20 | 0.17 | 1.05 | 0.89, 1.23 | 0.55 |
| MIP1a | 5 | 0.97 | 0.82, 1.14 | 0.72 | 0.98 | 0.72 | 0.39, 1.32 | 0.29 | 0.32 | 0.95 | 0.77, 1.16 | 0.59 |
| MIP1b | 31 | 0.92 | 0.86, 0.98 | 0.01 | 0.61 | 0.86 | 0.76, 0.96 | 0.01 | 0.14 | 0.91 | 0.85, 0.98 | 0.01 |
| PDGFbb | 6 | 0.99 | 0.85, 1.15 | 0.91 | 0.98 | 0.98 | 0.69, 1.39 | 0.91 | 0.94 | 1.00 | 0.88, 1.12 | 0.94 |
| RANTES | 8 | 1.03 | 0.89, 1.20 | 0.67 | 0.98 | 0.87 | 0.57, 1.32 | 0.51 | 0.39 | 1.02 | 0.87, 1.20 | 0.80 |
| SCF | 2 | 1.20 | 0.93, 1.55 | 0.15 | 0.90 | - | - | - | - | - | - | - |
| SCGFb | 4 | 1.07 | 0.88, 1.30 | 0.48 | 0.98 | 0.78 | 0.59, 1.03 | 0.08 | 0.01 | 1.08 | 0.93, 1.26 | 0.32 |
| SDF1a | 7 | 0.86 | 0.74, 1.02 | 0.08 | 0.90 | 0.77 | 0.60, 0.99 | 0.04 | 0.23 | 0.80 | 0.64, 0.99 | 0.04 |
| TNFa | 3 | 0.94 | 0.81, 1.08 | 0.37 | 0.94 | 0.91 | 0.66, 1.25 | 0.55 | 0.84 | 0.92 | 0.78, 1.09 | 0.34 |
| TNFb | 2 | 1.05 | 0.99, 1.13 | 0.13 | 0.90 | - | - | - | - | - | - | - |
| TRAIL | 12 | 1.01 | 0.95, 1.08 | 0.74 | 0.98 | 1.04 | 0.94, 1.14 | 0.44 | 0.46 | 1.01 | 0.93, 1.10 | 0.75 |
| VEGF | 14 | 1.01 | 0.95, 1.07 | 0.83 | 0.98 | 1.07 | 0.95, 1.22 | 0.26 | 0.24 | 1.03 | 0.95, 1.12 | 0.47 |

Table S8 Effects of any COVID-19 on 41 circulating cytokines using Mendelian Randomization with different methods (leave out 23 and me).

| Outcome | No. SNP | IVW |  |  |  | MR-Egger |  |  |  | WM |  |  |
| --- | --- | --- | --- | --- | --- | --- | --- | --- | --- | --- | --- | --- |
|  |  | Beta | 95% CI | P value | P_corrected | Beta | 95% CI | P value | P_intercept | Beta | 95% CI | P value |
| CTACK | 8 | 0.03 | -0.35, 0.41 | 0.86 | 0.94 | 0.03 | -1.01, 1.07 | 0.96 | 0.99 | 0.15 | -0.21, 0.51 | 0.42 |
| bNGF | 8 | 0.04 | -0.31, 0.39 | 0.84 | 0.94 | 0.56 | -0.28, 1.41 | 0.19 | 0.18 | 0.05 | -0.35, 0.44 | 0.81 |
| Eotaxin | 8 | -0.09 | -0.27, 0.08 | 0.29 | 0.70 | -0.17 | -0.61, 0.27 | 0.46 | 0.72 | -0.08 | -0.30, 0.14 | 0.48 |
| FGFBasic | 8 | -0.13 | -0.31, 0.05 | 0.15 | 0.43 | -0.13 | -0.59, 0.33 | 0.58 | 0.98 | -0.21 | -0.45, 0.03 | 0.08 |
| GCSF | 8 | -0.20 | -0.38, -0.02 | 0.03 | 0.23 | -0.46 | -0.91, -0.01 | 0.04 | 0.21 | -0.19 | -0.42, 0.05 | 0.12 |
| GROa | 8 | -0.22 | -0.49, 0.05 | 0.11 | 0.38 | -0.21 | -0.93, 0.51 | 0.57 | 0.98 | -0.27 | -0.62, 0.08 | 0.12 |
| HGF | 8 | -0.20 | -0.37, -0.03 | 0.02 | 0.23 | 0.09 | -0.35, 0.52 | 0.70 | 0.16 | -0.17 | -0.39, 0.05 | 0.13 |
| IFNg | 8 | -0.05 | -0.30, 0.19 | 0.66 | 0.92 | 0.07 | -0.58, 0.72 | 0.84 | 0.69 | -0.08 | -0.34, 0.18 | 0.56 |
| IL10 | 8 | -0.08 | -0.27, 0.10 | 0.38 | 0.82 | 0.33 | -0.12, 0.79 | 0.15 | 0.05 | -0.03 | -0.26, 0.21 | 0.83 |
| IP10 | 8 | 0.11 | -0.23, 0.45 | 0.53 | 0.85 | 0.83 | 0.11, 1.54 | 0.02 | 0.03 | 0.20 | -0.16, 0.56 | 0.27 |
| IL12p70 | 8 | -0.12 | -0.30, 0.05 | 0.16 | 0.43 | 0.00 | -0.44, 0.44 | 0.99 | 0.53 | -0.13 | -0.36, 0.10 | 0.26 |
| IL13 | 8 | -0.01 | -0.27, 0.26 | 0.95 | 0.96 | 0.28 | -0.40, 0.96 | 0.42 | 0.37 | 0.03 | -0.33, 0.38 | 0.88 |
| IL16 | 8 | 0.01 | -0.26, 0.28 | 0.93 | 0.96 | 0.31 | -0.38, 1.01 | 0.38 | 0.36 | -0.02 | -0.37, 0.33 | 0.92 |
| IL17 | 8 | -0.17 | -0.35, 0.01 | 0.07 | 0.29 | -0.18 | -0.63, 0.27 | 0.43 | 0.96 | -0.15 | -0.38, 0.08 | 0.21 |
| IL18 | 8 | -0.13 | -0.40, 0.13 | 0.31 | 0.72 | -0.48 | -1.14, 0.19 | 0.16 | 0.28 | -0.04 | -0.39, 0.31 | 0.84 |
| IL1b | 8 | -0.17 | -0.38, 0.04 | 0.12 | 0.38 | 0.05 | -0.50, 0.59 | 0.86 | 0.40 | -0.11 | -0.37, 0.16 | 0.43 |
| IL1ra | 8 | 0.17 | -0.12, 0.46 | 0.25 | 0.65 | 0.54 | -0.20, 1.27 | 0.15 | 0.29 | 0.24 | -0.12, 0.59 | 0.20 |
| IL2 | 7 | 0.12 | -0.20, 0.44 | 0.47 | 0.85 | 0.20 | -0.73, 1.12 | 0.68 | 0.86 | 0.07 | -0.32, 0.46 | 0.74 |
| IL2ra | 8 | -0.28 | -0.54, -0.01 | 0.04 | 0.25 | -0.82 | -1.48, -0.15 | 0.02 | 0.08 | -0.26 | -0.60, 0.08 | 0.14 |
| IL4 | 8 | -0.05 | -0.29, 0.18 | 0.67 | 0.92 | 0.05 | -0.58, 0.68 | 0.88 | 0.73 | -0.12 | -0.40, 0.15 | 0.37 |
| IL5 | 8 | -0.10 | -0.42, 0.23 | 0.56 | 0.85 | 0.04 | -0.84, 0.91 | 0.94 | 0.75 | 0.09 | -0.28, 0.45 | 0.64 |
| IL6 | 8 | 0.01 | -0.22, 0.23 | 0.96 | 0.96 | 0.33 | -0.22, 0.88 | 0.23 | 0.20 | -0.12 | -0.38, 0.14 | 0.38 |
| IL7 | 8 | -0.10 | -0.37, 0.18 | 0.49 | 0.85 | -0.03 | -0.72, 0.66 | 0.94 | 0.83 | -0.19 | -0.54, 0.16 | 0.28 |
| IL8 | 8 | -0.08 | -0.35, 0.18 | 0.54 | 0.85 | 0.01 | -0.67, 0.69 | 0.98 | 0.78 | -0.05 | -0.38, 0.28 | 0.77 |
| IL9 | 8 | 0.05 | -0.25, 0.35 | 0.73 | 0.92 | -0.22 | -1.00, 0.57 | 0.59 | 0.47 | 0.17 | -0.19, 0.53 | 0.35 |
| MCP1 | 7 | 0.13 | -0.22, 0.48 | 0.47 | 0.85 | 0.60 | -0.32, 1.52 | 0.20 | 0.28 | 0.09 | -0.17, 0.35 | 0.52 |
| MCP3 | 7 | 0.59 | 0.10, 1.08 | 0.02 | 0.23 | 1.53 | 0.24, 2.82 | 0.02 | 0.12 | 0.65 | 0.02, 1.27 | 0.04 |
| MCSF | 6 | -0.36 | -0.71, -0.01 | 0.04 | 0.25 | -0.14 | -1.12, 0.84 | 0.78 | 0.64 | -0.29 | -0.71, 0.13 | 0.17 |
| MIF | 8 | 0.07 | -0.20, 0.33 | 0.63 | 0.92 | 0.69 | 0.00, 1.37 | 0.05 | 0.05 | 0.13 | -0.21, 0.48 | 0.45 |
| MIG | 8 | -0.10 | -0.43, 0.23 | 0.56 | 0.85 | 0.49 | -0.26, 1.25 | 0.20 | 0.09 | -0.07 | -0.42, 0.29 | 0.71 |
| MIP1a | 8 | 0.04 | -0.22, 0.31 | 0.74 | 0.92 | 0.19 | -0.48, 0.87 | 0.57 | 0.64 | 0.16 | -0.18, 0.50 | 0.35 |
| MIP1b | 8 | -0.33 | -0.71, 0.05 | 0.09 | 0.35 | -1.14 | -1.90, -0.37 | 0.00 | 0.02 | -0.19 | -0.49, 0.11 | 0.22 |
| PDGFbb | 8 | -0.03 | -0.24, 0.18 | 0.78 | 0.94 | -0.12 | -0.68, 0.43 | 0.67 | 0.73 | 0.10 | -0.13, 0.33 | 0.40 |
| RANTES | 8 | 0.07 | -0.28, 0.42 | 0.71 | 0.92 | 0.75 | -0.01, 1.50 | 0.05 | 0.06 | 0.10 | -0.27, 0.47 | 0.60 |
| SCF | 8 | -0.16 | -0.33, 0.01 | 0.07 | 0.29 | 0.13 | -0.30, 0.57 | 0.55 | 0.15 | -0.21 | -0.44, 0.02 | 0.08 |
| SCGFb | 8 | -0.03 | -0.43, 0.37 | 0.87 | 0.94 | 0.76 | -0.09, 1.61 | 0.08 | 0.05 | 0.08 | -0.26, 0.43 | 0.64 |
| SDF1a | 8 | -0.07 | -0.25, 0.11 | 0.45 | 0.85 | -0.21 | -0.68, 0.27 | 0.40 | 0.54 | 0.04 | -0.21, 0.29 | 0.75 |
| TNFa | 8 | 0.04 | -0.30, 0.38 | 0.81 | 0.94 | 0.28 | -0.64, 1.19 | 0.55 | 0.59 | 0.23 | -0.13, 0.59 | 0.21 |
| TNFb | 6 | -0.52 | -0.95, -0.08 | 0.02 | 0.23 | -1.21 | -2.44, 0.01 | 0.05 | 0.23 | -0.63 | -1.17, -0.09 | 0.02 |
| TRAIL | 8 | -0.27 | -0.50, -0.03 | 0.03 | 0.23 | -0.13 | -0.76, 0.49 | 0.67 | 0.66 | -0.10 | -0.37, 0.16 | 0.44 |
| VEGF | 8 | -0.19 | -0.37, 0.00 | 0.05 | 0.26 | -0.06 | -0.53, 0.41 | 0.80 | 0.57 | -0.16 | -0.40, 0.08 | 0.20 |

Table S9 Effects of hospitalized COVID-19 on 41 circulating cytokines using Mendelian Randomization with different methods (leave out 23 and me).

| Outcome | No. SNP | IVW |  |  |  | MR-Egger |  |  |  | WM |  |  |
| --- | --- | --- | --- | --- | --- | --- | --- | --- | --- | --- | --- | --- |
|  |  | Beta | 95% CI | P value | P_corrected | Beta | 95% CI | P value | P_intercept | Beta | 95% CI | P value |
| CTACK | 8 | -0.07 | -0.21, 0.07 | 0.31 | 0.70 | -0.15 | -0.41, 0.11 | 0.27 | 0.50 | -0.08 | -0.23, 0.07 | 0.32 |
| bNGF | 8 | 0.01 | -0.17, 0.18 | 0.94 | 0.99 | 0.00 | -0.35, 0.35 | 0.99 | 0.97 | -0.01 | -0.16, 0.14 | 0.94 |
| Eotaxin | 8 | -0.06 | -0.14, 0.02 | 0.12 | 0.70 | -0.03 | -0.18, 0.11 | 0.64 | 0.65 | -0.05 | -0.15, 0.05 | 0.32 |
| FGFBasic | 8 | -0.05 | -0.13, 0.04 | 0.31 | 0.70 | -0.01 | -0.19, 0.16 | 0.87 | 0.67 | -0.06 | -0.16, 0.05 | 0.29 |
| GCSF | 8 | -0.07 | -0.15, 0.01 | 0.10 | 0.70 | -0.08 | -0.22, 0.07 | 0.31 | 0.89 | -0.09 | -0.19, 0.01 | 0.07 |
| GROa | 8 | -0.09 | -0.24, 0.06 | 0.26 | 0.70 | 0.04 | -0.24, 0.31 | 0.79 | 0.30 | -0.08 | -0.23, 0.07 | 0.31 |
| HGF | 8 | -0.05 | -0.16, 0.07 | 0.44 | 0.73 | 0.05 | -0.16, 0.26 | 0.62 | 0.27 | -0.03 | -0.12, 0.07 | 0.62 |
| IFNg | 8 | 0.01 | -0.07, 0.09 | 0.81 | 0.95 | -0.05 | -0.20, 0.09 | 0.47 | 0.31 | 0.02 | -0.09, 0.12 | 0.75 |
| IL10 | 8 | -0.03 | -0.13, 0.06 | 0.51 | 0.77 | 0.04 | -0.14, 0.22 | 0.66 | 0.34 | -0.03 | -0.13, 0.08 | 0.62 |
| IP10 | 8 | 0.07 | -0.07, 0.20 | 0.34 | 0.70 | 0.21 | -0.02, 0.44 | 0.07 | 0.14 | 0.13 | -0.02, 0.28 | 0.10 |
| IL12p70 | 8 | -0.05 | -0.13, 0.03 | 0.20 | 0.70 | -0.08 | -0.22, 0.07 | 0.29 | 0.68 | -0.06 | -0.16, 0.03 | 0.21 |
| IL13 | 8 | -0.03 | -0.16, 0.10 | 0.67 | 0.85 | -0.05 | -0.31, 0.20 | 0.67 | 0.81 | -0.04 | -0.19, 0.11 | 0.63 |
| IL16 | 8 | 0.05 | -0.07, 0.17 | 0.39 | 0.73 | 0.15 | -0.07, 0.38 | 0.18 | 0.29 | 0.07 | -0.08, 0.22 | 0.38 |
| IL17 | 8 | -0.05 | -0.13, 0.03 | 0.23 | 0.70 | -0.03 | -0.18, 0.12 | 0.72 | 0.73 | -0.06 | -0.16, 0.04 | 0.23 |
| IL18 | 8 | -0.10 | -0.22, 0.02 | 0.09 | 0.70 | -0.23 | -0.45, -0.01 | 0.04 | 0.19 | -0.15 | -0.31, 0.00 | 0.05 |
| IL1b | 8 | -0.04 | -0.18, 0.09 | 0.54 | 0.79 | 0.05 | -0.21, 0.30 | 0.71 | 0.41 | -0.04 | -0.16, 0.08 | 0.49 |
| IL1ra | 8 | 0.06 | -0.06, 0.18 | 0.34 | 0.70 | 0.03 | -0.21, 0.27 | 0.82 | 0.76 | 0.05 | -0.10, 0.20 | 0.53 |
| IL2 | 8 | 0.06 | -0.10, 0.22 | 0.46 | 0.73 | -0.01 | -0.32, 0.30 | 0.96 | 0.61 | 0.04 | -0.12, 0.19 | 0.64 |
| IL2ra | 8 | -0.15 | -0.29, -0.01 | 0.04 | 0.70 | -0.14 | -0.42, 0.13 | 0.31 | 0.95 | -0.16 | -0.31, -0.01 | 0.04 |
| IL4 | 8 | -0.07 | -0.17, 0.02 | 0.12 | 0.70 | -0.14 | -0.31, 0.03 | 0.11 | 0.36 | -0.11 | -0.21, -0.01 | 0.02 |
| IL5 | 8 | 0.01 | -0.14, 0.15 | 0.91 | 0.99 | 0.07 | -0.21, 0.35 | 0.62 | 0.60 | 0.03 | -0.12, 0.19 | 0.70 |
| IL6 | 8 | -0.02 | -0.10, 0.06 | 0.65 | 0.85 | -0.04 | -0.20, 0.13 | 0.66 | 0.80 | -0.04 | -0.14, 0.05 | 0.39 |
| IL7 | 8 | -0.05 | -0.17, 0.08 | 0.46 | 0.73 | -0.11 | -0.33, 0.12 | 0.36 | 0.54 | -0.07 | -0.23, 0.08 | 0.33 |
| IL8 | 8 | 0.00 | -0.12, 0.12 | 0.99 | 0.99 | 0.01 | -0.23, 0.25 | 0.92 | 0.91 | -0.04 | -0.19, 0.11 | 0.61 |
| IL9 | 8 | 0.01 | -0.11, 0.13 | 0.83 | 0.95 | -0.05 | -0.27, 0.17 | 0.68 | 0.53 | -0.02 | -0.18, 0.13 | 0.77 |
| MCP1 | 8 | 0.10 | 0.00, 0.21 | 0.06 | 0.70 | 0.25 | 0.10, 0.40 | 0.00 | 0.02 | 0.07 | -0.06, 0.19 | 0.30 |
| MCP3 | 7 | 0.17 | -0.08, 0.42 | 0.18 | 0.70 | 0.17 | -0.33, 0.67 | 0.51 | 0.99 | 0.29 | 0.01, 0.57 | 0.04 |
| MCSF | 8 | -0.13 | -0.31, 0.06 | 0.17 | 0.70 | 0.00 | -0.34, 0.34 | 0.99 | 0.39 | -0.09 | -0.27, 0.09 | 0.35 |
| MIF | 8 | 0.00 | -0.12, 0.13 | 0.95 | 0.99 | 0.02 | -0.21, 0.24 | 0.89 | 0.90 | 0.03 | -0.12, 0.18 | 0.70 |
| MIG | 8 | -0.02 | -0.18, 0.15 | 0.83 | 0.95 | 0.14 | -0.14, 0.43 | 0.32 | 0.18 | 0.06 | -0.09, 0.21 | 0.44 |
| MIP1a | 8 | 0.03 | -0.09, 0.15 | 0.66 | 0.85 | 0.05 | -0.18, 0.27 | 0.68 | 0.84 | 0.05 | -0.10, 0.20 | 0.52 |
| MIP1b | 8 | -0.06 | -0.30, 0.18 | 0.63 | 0.85 | -0.39 | -0.74, -0.04 | 0.03 | 0.03 | -0.20 | -0.37, -0.03 | 0.02 |
| PDGFbb | 8 | 0.01 | -0.07, 0.09 | 0.76 | 0.95 | -0.05 | -0.19, 0.09 | 0.51 | 0.32 | 0.03 | -0.07, 0.13 | 0.57 |
| RANTES | 8 | 0.08 | -0.12, 0.28 | 0.44 | 0.73 | 0.15 | -0.24, 0.55 | 0.44 | 0.66 | 0.09 | -0.07, 0.25 | 0.26 |
| SCF | 8 | -0.03 | -0.11, 0.05 | 0.46 | 0.73 | 0.04 | -0.11, 0.19 | 0.58 | 0.25 | -0.03 | -0.13, 0.07 | 0.56 |
| SCGFb | 8 | -0.08 | -0.21, 0.06 | 0.28 | 0.70 | 0.07 | -0.17, 0.30 | 0.58 | 0.16 | 0.01 | -0.16, 0.18 | 0.93 |
| SDF1a | 8 | -0.04 | -0.12, 0.04 | 0.34 | 0.70 | -0.15 | -0.30, 0.00 | 0.05 | 0.09 | -0.06 | -0.16, 0.05 | 0.29 |
| TNFa | 8 | 0.00 | -0.16, 0.16 | 0.98 | 0.99 | 0.04 | -0.27, 0.36 | 0.79 | 0.74 | 0.01 | -0.14, 0.17 | 0.87 |
| TNFb | 6 | -0.20 | -0.40, -0.01 | 0.04 | 0.70 | -0.34 | -0.70, 0.01 | 0.06 | 0.35 | -0.25 | -0.48, -0.03 | 0.03 |
| TRAIL | 8 | -0.08 | -0.20, 0.05 | 0.23 | 0.70 | 0.11 | -0.07, 0.29 | 0.24 | 0.02 | -0.02 | -0.12, 0.08 | 0.66 |
| VEGF | 8 | -0.06 | -0.15, 0.02 | 0.14 | 0.70 | 0.00 | -0.15, 0.16 | 0.99 | 0.33 | -0.05 | -0.15, 0.06 | 0.37 |

Table S10 Effects of any COVID-19 on 41 circulating cytokines using Mendelian Randomization with different methods (leave out ukb).

| Outcome | No. SNP | IVW |  |  |  | MR-Egger |  |  |  | WM |  |  |
| --- | --- | --- | --- | --- | --- | --- | --- | --- | --- | --- | --- | --- |
|  |  | Beta | 95% CI | P value | P_corrected | Beta | 95% CI | P value | P_intercept | Beta | 95% CI | P value |
| CTACK | 5 | -0.15 | -0.56, 0.26 | 0.48 | 0.88 | -0.16 | -1.34, 1.02 | 0.79 | 0.98 | -0.08 | -0.46, 0.29 | 0.66 |
| bNGF | 5 | 0.04 | -0.43, 0.51 | 0.88 | 0.94 | 0.39 | -0.90, 1.68 | 0.55 | 0.56 | 0.02 | -0.38, 0.43 | 0.91 |
| Eotaxin | 5 | 0.00 | -0.19, 0.19 | 0.98 | 0.98 | -0.03 | -0.50, 0.44 | 0.91 | 0.91 | 0.00 | -0.23, 0.22 | 0.99 |
| FGFBasic | 5 | -0.08 | -0.28, 0.12 | 0.42 | 0.88 | -0.04 | -0.53, 0.45 | 0.86 | 0.87 | -0.12 | -0.36, 0.12 | 0.32 |
| GCSF | 5 | -0.16 | -0.35, 0.03 | 0.10 | 0.47 | -0.42 | -0.90, 0.06 | 0.08 | 0.24 | -0.16 | -0.39, 0.08 | 0.20 |
| GROa | 5 | -0.37 | -0.66, -0.08 | 0.01 | 0.20 | -0.05 | -0.77, 0.67 | 0.90 | 0.34 | -0.35 | -0.71, 0.00 | 0.05 |
| HGF | 5 | -0.15 | -0.34, 0.04 | 0.11 | 0.47 | 0.05 | -0.42, 0.52 | 0.83 | 0.36 | -0.08 | -0.31, 0.15 | 0.49 |
| IFNg | 5 | 0.02 | -0.20, 0.24 | 0.86 | 0.94 | -0.03 | -0.66, 0.60 | 0.93 | 0.87 | 0.04 | -0.22, 0.30 | 0.75 |
| IL10 | 5 | -0.06 | -0.26, 0.13 | 0.52 | 0.88 | 0.16 | -0.33, 0.65 | 0.52 | 0.33 | -0.02 | -0.26, 0.22 | 0.87 |
| IP10 | 5 | 0.24 | -0.25, 0.73 | 0.34 | 0.76 | 1.04 | 0.01, 2.07 | 0.05 | 0.10 | 0.42 | 0.05, 0.78 | 0.03 |
| IL12p70 | 5 | -0.10 | -0.29, 0.09 | 0.30 | 0.76 | -0.13 | -0.63, 0.36 | 0.59 | 0.88 | -0.08 | -0.31, 0.16 | 0.54 |
| IL13 | 5 | 0.01 | -0.29, 0.31 | 0.95 | 0.98 | 0.25 | -0.57, 1.08 | 0.55 | 0.53 | 0.01 | -0.37, 0.38 | 0.97 |
| IL16 | 5 | 0.07 | -0.22, 0.35 | 0.66 | 0.90 | 0.04 | -0.70, 0.79 | 0.91 | 0.95 | 0.07 | -0.28, 0.43 | 0.67 |
| IL17 | 5 | -0.12 | -0.31, 0.08 | 0.24 | 0.72 | -0.18 | -0.66, 0.31 | 0.47 | 0.79 | -0.17 | -0.40, 0.06 | 0.15 |
| IL18 | 5 | -0.20 | -0.52, 0.12 | 0.21 | 0.72 | -0.93 | -1.64, -0.23 | 0.01 | 0.03 | -0.25 | -0.63, 0.13 | 0.20 |
| IL1b | 5 | -0.21 | -0.43, 0.02 | 0.07 | 0.40 | -0.03 | -0.59, 0.54 | 0.93 | 0.49 | -0.13 | -0.41, 0.15 | 0.36 |
| IL1ra | 5 | 0.19 | -0.19, 0.57 | 0.33 | 0.76 | 0.68 | -0.24, 1.61 | 0.15 | 0.25 | 0.21 | -0.16, 0.59 | 0.27 |
| IL2 | 5 | 0.13 | -0.24, 0.50 | 0.50 | 0.88 | 0.39 | -0.63, 1.41 | 0.46 | 0.59 | 0.13 | -0.26, 0.52 | 0.51 |
| IL2ra | 5 | -0.37 | -0.65, -0.09 | 0.01 | 0.20 | -0.77 | -1.47, -0.06 | 0.03 | 0.23 | -0.40 | -0.75, -0.05 | 0.02 |
| IL4 | 5 | -0.06 | -0.36, 0.24 | 0.69 | 0.90 | -0.24 | -1.08, 0.60 | 0.58 | 0.65 | -0.22 | -0.49, 0.06 | 0.12 |
| IL5 | 5 | -0.05 | -0.44, 0.33 | 0.78 | 0.93 | 0.42 | -0.52, 1.36 | 0.38 | 0.28 | 0.06 | -0.32, 0.44 | 0.76 |
| IL6 | 5 | 0.04 | -0.18, 0.26 | 0.70 | 0.90 | 0.08 | -0.56, 0.72 | 0.81 | 0.91 | -0.01 | -0.26, 0.24 | 0.95 |
| IL7 | 5 | -0.08 | -0.37, 0.21 | 0.61 | 0.88 | 0.05 | -0.67, 0.78 | 0.89 | 0.70 | -0.08 | -0.45, 0.28 | 0.66 |
| IL8 | 5 | -0.17 | -0.46, 0.12 | 0.25 | 0.72 | -0.22 | -0.93, 0.50 | 0.56 | 0.89 | -0.09 | -0.44, 0.26 | 0.60 |
| IL9 | 5 | 0.04 | -0.31, 0.39 | 0.82 | 0.93 | 0.18 | -0.82, 1.18 | 0.73 | 0.77 | 0.06 | -0.29, 0.42 | 0.72 |
| MCP1 | 5 | 0.20 | -0.15, 0.55 | 0.27 | 0.74 | 0.59 | -0.30, 1.48 | 0.20 | 0.35 | 0.22 | -0.07, 0.52 | 0.14 |
| MCP3 | 4 | 0.61 | 0.06, 1.16 | 0.03 | 0.24 | 1.11 | -0.37, 2.59 | 0.14 | 0.48 | 0.63 | 0.00, 1.27 | 0.05 |
| MCSF | 5 | -0.41 | -0.75, -0.07 | 0.02 | 0.20 | -0.53 | -1.41, 0.35 | 0.24 | 0.77 | -0.28 | -0.69, 0.14 | 0.19 |
| MIF | 5 | 0.08 | -0.21, 0.37 | 0.57 | 0.88 | 0.22 | -0.51, 0.95 | 0.55 | 0.69 | 0.13 | -0.21, 0.47 | 0.46 |
| MIG | 5 | -0.07 | -0.53, 0.38 | 0.75 | 0.93 | 0.68 | -0.23, 1.60 | 0.14 | 0.08 | 0.13 | -0.24, 0.49 | 0.51 |
| MIP1a | 5 | 0.09 | -0.20, 0.38 | 0.54 | 0.88 | 0.27 | -0.44, 0.99 | 0.45 | 0.58 | 0.14 | -0.21, 0.49 | 0.43 |
| MIP1b | 5 | -0.40 | -0.83, 0.02 | 0.06 | 0.40 | -1.29 | -1.87, -0.70 | 0.00 | 0.00 | -0.36 | -0.69, -0.03 | 0.03 |
| PDGFbb | 5 | 0.05 | -0.14, 0.24 | 0.60 | 0.88 | -0.29 | -0.76, 0.18 | 0.23 | 0.12 | 0.07 | -0.17, 0.31 | 0.58 |
| RANTES | 5 | 0.10 | -0.19, 0.39 | 0.49 | 0.88 | 0.39 | -0.37, 1.14 | 0.32 | 0.42 | 0.02 | -0.36, 0.40 | 0.91 |
| SCF | 5 | -0.07 | -0.28, 0.13 | 0.48 | 0.88 | 0.29 | -0.17, 0.76 | 0.22 | 0.09 | -0.13 | -0.38, 0.12 | 0.32 |
| SCGFb | 5 | -0.04 | -0.35, 0.26 | 0.79 | 0.93 | 0.35 | -0.39, 1.09 | 0.36 | 0.26 | 0.09 | -0.27, 0.44 | 0.63 |
| SDF1a | 5 | -0.05 | -0.27, 0.16 | 0.62 | 0.88 | -0.38 | -0.86, 0.11 | 0.13 | 0.15 | 0.01 | -0.25, 0.28 | 0.91 |
| TNFa | 5 | 0.02 | -0.37, 0.41 | 0.93 | 0.98 | 0.58 | -0.31, 1.48 | 0.20 | 0.18 | 0.15 | -0.23, 0.53 | 0.44 |
| TNFb | 4 | -0.55 | -1.01, -0.09 | 0.02 | 0.20 | -1.19 | -2.42, 0.03 | 0.06 | 0.27 | -0.67 | -1.23, -0.12 | 0.02 |
| TRAIL | 5 | -0.27 | -0.60, 0.05 | 0.10 | 0.47 | -0.09 | -1.01, 0.82 | 0.84 | 0.67 | -0.28 | -0.56, 0.00 | 0.05 |
| VEGF | 5 | -0.14 | -0.35, 0.06 | 0.16 | 0.61 | -0.02 | -0.52, 0.49 | 0.95 | 0.59 | -0.13 | -0.38, 0.11 | 0.28 |

Table S11 Effects of hospitalized COVID-19 on 41 circulating cytokines using Mendelian Randomization with different methods (leave out ukb).

| Outcome | No. SNP | IVW |  |  |  | MR-Egger |  |  |  | WM |  |  |
| --- | --- | --- | --- | --- | --- | --- | --- | --- | --- | --- | --- | --- |
|  |  | Beta | 95% CI | P value | P_corrected | Beta | 95% CI | P value | P_intercept | Beta | 95% CI | P value |
| CTACK | 4 | -0.10 | -0.26, 0.07 | 0.26 | 0.52 | -0.12 | -0.49, 0.26 | 0.54 | 0.88 | -0.07 | -0.22, 0.07 | 0.31 |
| bNGF | 4 | -0.06 | -0.20, 0.09 | 0.43 | 0.69 | 0.11 | -0.12, 0.33 | 0.34 | 0.08 | -0.02 | -0.16, 0.11 | 0.76 |
| Eotaxin | 4 | -0.04 | -0.12, 0.04 | 0.30 | 0.55 | -0.05 | -0.20, 0.10 | 0.50 | 0.89 | -0.04 | -0.13, 0.05 | 0.35 |
| FGFBasic | 4 | -0.05 | -0.13, 0.03 | 0.24 | 0.52 | -0.07 | -0.22, 0.09 | 0.39 | 0.79 | -0.06 | -0.15, 0.03 | 0.19 |
| GCSF | 4 | -0.05 | -0.13, 0.03 | 0.19 | 0.52 | -0.13 | -0.27, 0.02 | 0.09 | 0.26 | -0.07 | -0.16, 0.02 | 0.12 |
| GROa | 4 | -0.05 | -0.22, 0.12 | 0.57 | 0.77 | -0.01 | -0.39, 0.37 | 0.96 | 0.81 | -0.05 | -0.19, 0.09 | 0.45 |
| HGF | 4 | -0.04 | -0.12, 0.03 | 0.27 | 0.52 | 0.04 | -0.11, 0.18 | 0.62 | 0.19 | -0.02 | -0.11, 0.07 | 0.67 |
| IFNg | 4 | -0.02 | -0.10, 0.06 | 0.58 | 0.77 | -0.03 | -0.19, 0.12 | 0.68 | 0.87 | -0.02 | -0.11, 0.07 | 0.72 |
| IL10 | 4 | -0.05 | -0.13, 0.03 | 0.22 | 0.52 | 0.02 | -0.13, 0.17 | 0.83 | 0.30 | -0.03 | -0.13, 0.06 | 0.49 |
| IP10 | 4 | 0.07 | -0.11, 0.26 | 0.43 | 0.69 | 0.26 | -0.03, 0.55 | 0.08 | 0.14 | 0.13 | 0.00, 0.27 | 0.05 |
| IL12p70 | 4 | -0.07 | -0.15, 0.01 | 0.09 | 0.52 | -0.09 | -0.23, 0.06 | 0.24 | 0.77 | -0.07 | -0.16, 0.02 | 0.13 |
| IL13 | 4 | -0.07 | -0.19, 0.05 | 0.22 | 0.52 | 0.04 | -0.18, 0.27 | 0.71 | 0.23 | -0.08 | -0.22, 0.05 | 0.22 |
| IL16 | 4 | 0.07 | -0.05, 0.20 | 0.23 | 0.52 | 0.12 | -0.10, 0.35 | 0.29 | 0.63 | 0.08 | -0.05, 0.22 | 0.22 |
| IL17 | 4 | -0.05 | -0.13, 0.03 | 0.20 | 0.52 | -0.06 | -0.21, 0.09 | 0.41 | 0.89 | -0.06 | -0.14, 0.03 | 0.22 |
| IL18 | 4 | -0.10 | -0.23, 0.03 | 0.14 | 0.52 | -0.25 | -0.47, -0.03 | 0.02 | 0.10 | -0.12 | -0.25, 0.02 | 0.09 |
| IL1b | 4 | -0.06 | -0.16, 0.03 | 0.20 | 0.52 | 0.05 | -0.12, 0.23 | 0.55 | 0.13 | -0.05 | -0.16, 0.05 | 0.33 |
| IL1ra | 4 | 0.01 | -0.11, 0.13 | 0.92 | 0.94 | 0.10 | -0.13, 0.32 | 0.40 | 0.35 | 0.03 | -0.10, 0.16 | 0.67 |
| IL2 | 4 | -0.02 | -0.14, 0.10 | 0.73 | 0.88 | 0.02 | -0.22, 0.25 | 0.90 | 0.72 | -0.01 | -0.15, 0.13 | 0.88 |
| IL2ra | 4 | -0.11 | -0.23, 0.01 | 0.06 | 0.52 | -0.15 | -0.38, 0.07 | 0.18 | 0.66 | -0.12 | -0.25, 0.01 | 0.08 |
| IL4 | 4 | -0.09 | -0.17, -0.01 | 0.02 | 0.52 | -0.12 | -0.26, 0.03 | 0.12 | 0.71 | -0.10 | -0.19, -0.02 | 0.02 |
| IL5 | 4 | -0.04 | -0.20, 0.12 | 0.62 | 0.79 | 0.13 | -0.10, 0.36 | 0.26 | 0.08 | 0.01 | -0.13, 0.15 | 0.90 |
| IL6 | 4 | -0.04 | -0.12, 0.03 | 0.26 | 0.52 | -0.02 | -0.16, 0.13 | 0.80 | 0.68 | -0.04 | -0.13, 0.04 | 0.33 |
| IL7 | 4 | -0.08 | -0.20, 0.05 | 0.22 | 0.52 | -0.06 | -0.29, 0.16 | 0.59 | 0.89 | -0.07 | -0.21, 0.06 | 0.28 |
| IL8 | 4 | -0.05 | -0.17, 0.07 | 0.37 | 0.66 | 0.01 | -0.21, 0.23 | 0.92 | 0.49 | -0.04 | -0.18, 0.09 | 0.51 |
| IL9 | 4 | -0.02 | -0.15, 0.11 | 0.77 | 0.89 | -0.07 | -0.36, 0.22 | 0.64 | 0.69 | -0.02 | -0.15, 0.12 | 0.79 |
| MCP1 | 4 | 0.09 | -0.04, 0.23 | 0.18 | 0.52 | 0.28 | 0.14, 0.43 | 0.00 | 0.00 | 0.08 | -0.01, 0.18 | 0.08 |
| MCP3 | 4 | 0.19 | -0.03, 0.41 | 0.09 | 0.52 | 0.36 | -0.04, 0.77 | 0.08 | 0.32 | 0.25 | 0.01, 0.50 | 0.04 |
| MCSF | 4 | -0.12 | -0.26, 0.03 | 0.11 | 0.52 | -0.01 | -0.28, 0.26 | 0.94 | 0.35 | -0.07 | -0.24, 0.09 | 0.37 |
| MIF | 4 | 0.01 | -0.11, 0.13 | 0.90 | 0.94 | 0.11 | -0.12, 0.33 | 0.35 | 0.31 | 0.03 | -0.11, 0.16 | 0.71 |
| MIG | 4 | 0.00 | -0.21, 0.20 | 0.99 | 0.99 | 0.18 | -0.17, 0.53 | 0.30 | 0.22 | 0.05 | -0.08, 0.19 | 0.42 |
| MIP1a | 4 | -0.01 | -0.13, 0.11 | 0.87 | 0.94 | 0.11 | -0.12, 0.33 | 0.34 | 0.22 | 0.03 | -0.11, 0.16 | 0.69 |
| MIP1b | 4 | -0.14 | -0.33, 0.04 | 0.13 | 0.52 | -0.35 | -0.61, -0.09 | 0.01 | 0.06 | -0.20 | -0.32, -0.09 | 0.00 |
| PDGFbb | 4 | 0.01 | -0.07, 0.09 | 0.86 | 0.94 | -0.09 | -0.23, 0.06 | 0.24 | 0.13 | 0.00 | -0.08, 0.09 | 0.92 |
| RANTES | 4 | 0.04 | -0.17, 0.25 | 0.71 | 0.88 | 0.26 | -0.06, 0.57 | 0.11 | 0.11 | 0.09 | -0.05, 0.24 | 0.21 |
| SCF | 4 | -0.01 | -0.09, 0.07 | 0.78 | 0.89 | 0.07 | -0.08, 0.21 | 0.36 | 0.21 | -0.01 | -0.10, 0.07 | 0.74 |
| SCGFb | 4 | -0.07 | -0.27, 0.13 | 0.51 | 0.73 | 0.14 | -0.16, 0.45 | 0.35 | 0.10 | 0.03 | -0.12, 0.18 | 0.71 |
| SDF1a | 4 | -0.04 | -0.13, 0.05 | 0.38 | 0.66 | -0.17 | -0.31, -0.02 | 0.03 | 0.05 | -0.03 | -0.12, 0.06 | 0.53 |
| TNFa | 4 | -0.05 | -0.19, 0.10 | 0.51 | 0.73 | 0.11 | -0.12, 0.34 | 0.34 | 0.10 | 0.01 | -0.13, 0.14 | 0.92 |
| TNFb | 4 | -0.19 | -0.37, -0.01 | 0.04 | 0.52 | -0.34 | -0.67, 0.00 | 0.05 | 0.30 | -0.22 | -0.42, -0.02 | 0.03 |
| TRAIL | 4 | -0.03 | -0.11, 0.05 | 0.50 | 0.73 | 0.05 | -0.10, 0.19 | 0.53 | 0.23 | -0.01 | -0.10, 0.08 | 0.88 |
| VEGF | 4 | -0.06 | -0.15, 0.02 | 0.14 | 0.52 | -0.01 | -0.17, 0.14 | 0.89 | 0.44 | -0.04 | -0.14, 0.05 | 0.37 |

Table S18 Effects of any COVID-19 on macrophage inflammatory protein-1β (MIP1b) using Mendelian Randomization with different methods after removing instruments identified from Steiger filtering (leave out 23 and me).

| Outcome | No. SNP | IVW |  |  | MR-Egger |  |  |  | WM |  |  |
| --- | --- | --- | --- | --- | --- | --- | --- | --- | --- | --- | --- |
|  |  | Beta | 95% CI | P value | Beta | 95% CI | P value | P_intercept | Beta | 95% CI | P value |
| MIP1b | 6* | 0.02 | -0.19, 0.24 | 0.82 | 0.01 | -0.81, 0.83 | 0.98 | 0.97 | -0.03 | -0.30, 0.24 | 0.83 |

*Removing rs2271616 and rs35508621.


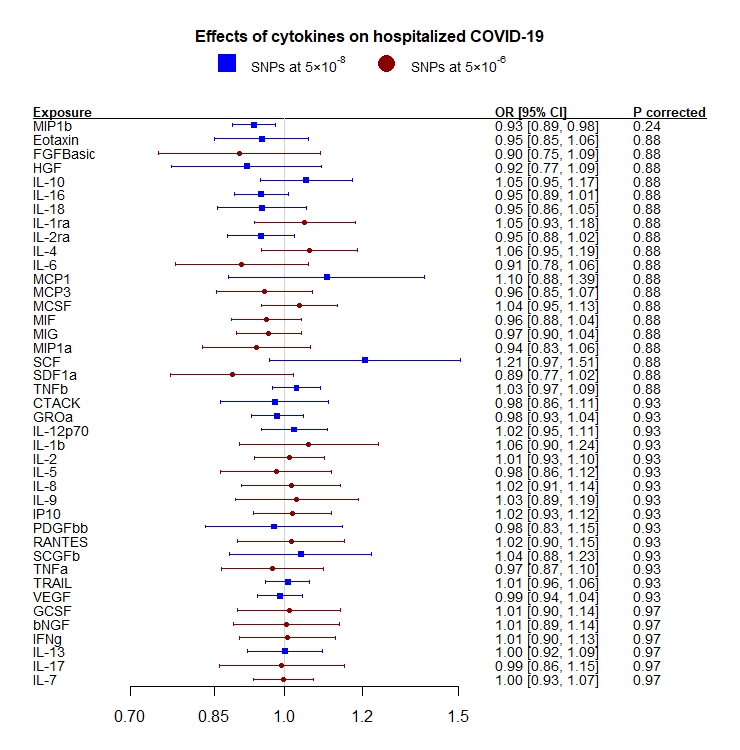


Supplementary Figure 1 Effects of 41 circulating cytokines on hospitalized COVID-19 using inverse variance weighting.


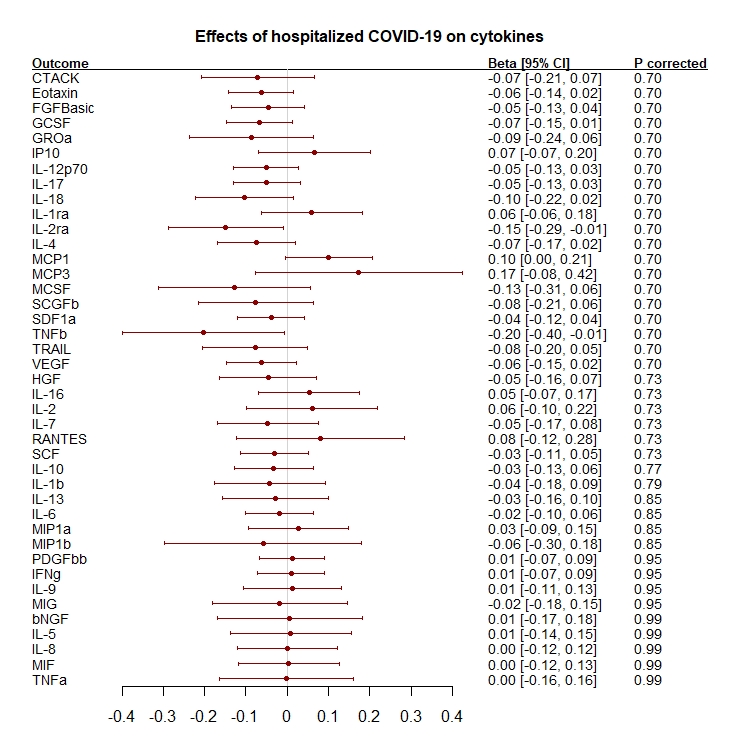
Supplementary Figure 2 Effects of hospitalized COVID-19 on 41 circulating cytokines using inverse variance weighting.
